# Inhibition of Enhancer of Zeste Homolog 2 Induces Blast Differentiation, Impairs Engraftment and Prolongs Survival in Murine Models of Acute Myeloid Leukemia

**DOI:** 10.3390/cancers16030569

**Published:** 2024-01-29

**Authors:** Sydney Fobare, Ola A. Elgamal, Mark Wunderlich, Emily Stahl, Abeera Mehmood, Casie Furby, James R. Lerma, Thomas M. Sesterhenn, Jianmin Pan, Jayesh Rai, Megan E. Johnstone, Amina Abdul-Aziz, Mariah L. Johnson, Shesh N. Rai, John C. Byrd, Erin Hertlein

**Affiliations:** 1 Medical Scientist Training Program, The Ohio State University, Columbus, OH 43210, USA; sydney.fobare@osumc.edu; 2 Department of Internal Medicine, University of Cincinnati, Cincinnati, OH 45229, USAbyrd2jc@ucmail.uc.edu (J.C.B.); 3 Experimental Hematology and Cancer Biology, Cincinnati Children’s Hospital, Cincinnati, OH 45229, USA; 4 Department of Internal Medicine, The Ohio State University, Columbus, OH 43210, USA; 5 Division of Biostatistics and Bioinformatics, Department of Environmental Health and Public Health Sciences, University of Cincinnati, Cincinnati, OH 45267, USA; 6 The Cancer Data Science Center, Department of Environmental Health and Public Health Sciences, University of Cincinnati, Cincinnati, OH 45267, USA; 7 Biostatistics and Informatics Shared Resource, University of Cincinnati Cancer Center, Cincinnati, OH 45267, USA

**Keywords:** EZH2, acute myeloid leukemia, differentiation therapy

## Abstract

**Simple Summary:**

Acute myeloid leukemia (AML) is the abnormal growth of immature blood cells characterized by a block in differentiation. The therapy options to treat AML have primarily consisted of cytotoxic chemotherapy, until more recently, when small-molecule targeted therapy emerged. Many of these targeted therapies are directed at agents that can cause abnormal cells to differentiate. Herein, we discuss the role of a small molecule inhibitor of enhancer of zeste homolog 2 (EZH2), which can induce differentiation and promote survival in AML models.

**Abstract:**

Background: Acute myeloid leukemia (AML) is the malignant proliferation of immature myeloid cells characterized by a block in differentiation. As such, novel therapeutic strategies to promote the differentiation of immature myeloid cells have been successful in AML, although these agents are targeted to a specific mutation that is only present in a subset of AML patients. In the current study, we show that targeting the epigenetic modifier enhancer of zeste homolog 2 (EZH2) can induce the differentiation of immature blast cells into a more mature myeloid phenotype and promote survival in AML murine models. Methods: The EZH2 inhibitor EPZ011989 (EPZ) was studied in AML cell lines, primary in AML cells and normal CD34+ stem cells. A pharmacodynamic assessment of H3K27me3; studies of differentiation, cell growth, and colony formation; and in vivo therapeutic studies including the influence on primary AML cell engraftment were also conducted. Results: EPZ inhibited H3K27me3 in AML cell lines and primary AML samples in vitro. EZH2 inhibition reduced colony formation in multiple AML cell lines and primary AML samples, while exhibiting no effect on colony formation in normal CD34+ stem cells. In AML cells, EPZ promoted phenotypic evidence of differentiation. Finally, the pretreatment of primary AML cells with EPZ significantly delayed engraftment and prolonged the overall survival when engrafted into immunodeficient mice. Conclusions: Despite evidence that EZH2 silencing in MDS/MPN can promote AML pathogenesis, our data demonstrate that the therapeutic inhibition of EZH2 in established AML has the potential to improve survival.

## 1. Introduction

Acute myeloid leukemia (AML) is the malignant proliferation of immature myeloid cells due to a block in differentiation [1]. It is the most commonly diagnosed acute leukemia in adults, and these patients have poor outcomes, with long-term cure rates of 30–40% in younger patients treated with induction and consolidation chemotherapy [2]. In patients over the age of 60, the results are even more unfavorable, as the chances of survival are below 10–15% when treated with aggressive chemotherapy [3,4]. Outside of select favorable genomic groups, AML is not curable in the absence of an allogeneic stem cell transplant (ASCT). However, the median age of AML diagnosis is 67, which makes ASCT irrelevant to most patients with this disease and provides a strong rationale for the application of different types of therapies in this disease. Mutations in FLT3, IDH1, and IDH2 encompass 25% of elderly patients with AML, and therapies directed at these mutations (gilteritinib [5], ivosidenib [6], and enasidenib [7], respectively) mediate differentiation as their primary mechanism of action. These agents can promote durable remission, but relapse eventually occurs due to the expansion of pre-existing clones lacking these mutations or the development of acquired resistance clones [8,9,10]. While these FDA-approved treatments have improved survival, most patients still do not obtain long-term durable remission [2]. Accordingly, the need for novel targeted therapies with minimal side effects that do not impact normal hematopoiesis are necessary.

The role of enhancer of zeste homolog 2 (EZH2) in blood cancers is not well understood. EZH2 is the catalytic core subunit of the polycomb repressive complex 2 (PRC2) and methylates lysine 27 on histone H3 (H3K27me3), resulting in transcriptional repression [11]. Recently, EZH2 inhibitors, such as tazemetostat, have been developed for clinical use in relapsed/refractory follicular lymphoma where *EZH2* mutations are typically gain-of-function [12]. Preclinical data support the use of tazemetostat in both *EZH2*-mutant and wild-type non-Hodgkin’s lymphoma, as tazemetostat can be cytotoxic and cytostatic, respectively [13]. These data provided the basis of a phase 2 trial and the subsequent FDA approval of tazemetostat in both *EZH2*-mutant and wild-type relapsed/refractory follicular lymphoma [14]. The promising results with tazemetostat in *EZH2* wild-type lymphoma support the exploration of EZH2 inhibition in other hematological malignancies. Unlike lymphoma, *EZH2* mutations in AML tend to be loss-of-function mutations [15], and studies indicate that loss-of-function EZH2 mutations in AML patients have poor prognoses and are associated with a reduced overall survival [16,17]. However, the role of EZH2 in AML can be context-dependent, as it has been shown to function as an oncogene during AML development and a tumor suppressor during the AML maintenance phase [18]. Basheer et al. demonstrated that EZH2 is regulating a different set of genes in normal murine bone marrow hematopoietic stem and progenitor cells compared to AML1-ETO9a murine leukemia cells [18]. We hypothesized that, in patients with *EZH2* wild-type AML, the inhibition of EZH2 may produce a phenotype that would allow for therapeutic targeting without influencing normal hematopoiesis. To study this, we used EPZ011989 [19] to inhibit EZH2 in AML cell lines as well as primary samples. It is important to understand the role of EZH2 in AML blast cells in order develop rational combination therapies that will target blasts without affecting normal hematopoiesis.

## 2. Materials and Methods

### 2.1. Chemical Compounds

EPZ011989 (EPZ) was purchased from Selleckchem (Houston, TX, USA, Cat# S7805).

### 2.2. Cell Lines and Culture Conditions

MOLM-13, OCI-AML3, and MV4-11 cells were purchased from the Leibniz Institute DSMZ-German Collection of Microorganisms and Cell Cultures GmbH. Our lab previously isolated a *TP53* wild-type MV4-11 subclone, which was used in subsequent experiments [20]. The MOLM-13 luciferase-expressing cell line was a generous gift from Dr. Ramiro Garzon [21]. Cells were cultured in Roswell Park Memorial Institute (RPMI) 1640 with 10% fetal bovine serum, 2 mM of L-glutamine, 56 U/mL of penicillin, and 56 µg/mL of streptomycin (Thermo Fisher Scientific, Waltham, MA, USA). Cell lines were routinely tested for mycoplasma and underwent short tandem repeat (STR) testing for authentication. Cells were incubated at 37 °C and 5% CO_2_.

### 2.3. Primary AML Samples and Culture Conditions

Primary AML samples (apheresis, bone marrow, and peripheral blood) were obtained from the Ohio State University Comprehensive Cancer Center Leukemia Tissue Bank (LTB). Samples were collected under an Institutional Review Board-approved protocol, and the LTB acted as an honest broker. Samples were cultured in StemSpan (StemCell Technologies, Cambridge, MA, USA) that was supplemented with Flt3-L, SCF, GM-CSF, IL-3, G-CSF, IL-6, TPO, and EPO (PeproTech, Cranbury, NJ, USA). Cells were incubated at 37 °C and 5% CO_2_.

### 2.4. Antibodies

Anti-H3K27me3 (Cat# 9733), anti-Histone H3 (Cat# 3638), anti-EZH2 (Cat# 5246), and anti-β-actin (Cat# 3700) antibodies were purchased from Cell Signaling Technology (Danvers, MA, USA) for immunoblots. β-Actin was used as a loading control.

### 2.5. Cell Treatment and Immunoblots

For immunoblot assays, MOLM-13, OCI-AML3, and MV4-11 cells were cultured with dimethyl sulfoxide (DMSO), 0.5 µM, or 1 µM EPZ for 96 h, and primary AML samples were treated with DMSO or 1 µM EPZ for 7 days. Cells were washed with PBS and then lysed in cell lysis buffer with protease inhibitor cocktail, serine/threonine phosphatase inhibitor cocktail, tyrosine phosphatase inhibitor, and phenylmethanesulfonyl fluoride solution (Millipore Sigma, Burlington, MA, USA). Samples were then sonicated to shear the nucleus. Pierce BCA Protein Assay (Thermo Fisher Scientific) was used to quantify protein lysate. An amount of 25 ug of protein lysate was loaded for the cell lines, and 20 ug of protein lysate was loaded for the primary samples. The cell line samples were run on a 12% sodium dodecyl sulfate polyacrylamide gel, and the primary AML samples were run on Mini-PROTEAN TGX Stain-Free Precast Gels (Bio-Rad, Hercules, CA, USA). Gels were transferred to a nitrocellulose membrane. Gels with cell line lysates were blocked for 1 h in Blocker BLOTTO in TBS (Thermo Fisher Scientific) and then incubated overnight at 4 °C with primary antibody. Blots were washed and incubated with horseradish peroxidase-conjugated secondary antibody for 1 h at room temperature and then developed with chemiluminescent Pierce ECL Substrate (Thermo Fisher Scientific) on X-ray films. Primary AML sample immunoblots were blocked for 1 h in Intercept (TBS) Blocking Buffer (Li-cor, Lincoln, NE, USA) and incubated overnight at 4 °C with primary antibody diluted in Intercept T20 (TBS) Antibody Diluent (Li-cor). Blots were washed and incubated with secondary antibody diluted in Intercept T20 (TBS) Antibody Diluent (Li-cor) for 1 h at room temperature. Proteins were detected with fluorescent imaging on the Odyssey M Imaging System (Li-cor).

### 2.6. Proliferation Assay

MOLM-13, OCI-AML3, or MV4-11 (3125 cells per well) and primary AML (100,000 cells per well) were plated in 96-well plates for 7 days. Plates were spun down, and the media were refreshed on day 4. On day 7, Cell Titer 96 Aqueous MTS Reagent Powder (Promega) and phenazine methosulfate (Millipore Sigma) were added. After a 3 h incubation period, absorbance was measured at 490 nm on the Cytation 5 Cell Imaging Multi-Mode Reader (Agilent, Santa Clara, CA, USA).

### 2.7. Colony Formation Unit (CFU) Assay

MOLM-13, OCI-AML3, or MV4-11 (156 cells per condition) were plated with DMSO or 1 µM EPZ in MethoCult H4035 Optimum Without EPO (StemCell Technologies, Cambridge, MA, USA) and incubated at 37 °C with 5% CO_2_ for 7 days. Images were taken with the STEMVision Instrument, and the colonies were counted manually. For primary AML samples, cells were plated at the appropriate density (5 × 10^4^–2 × 10^5^ cells/well) with DMSO or 1 µM EPZ in MethoCult H4035 Optimum Without EPO and incubated at 37 °C with 5% CO_2_ and 1% O_2_ for 14 days. Images were taken with the STEMVision Instrument, and colonies were counted using the appropriate STEMVision algorithm or manually by two counters blinded to experimental conditions on the Revolve Microscope (ECHO). Samples that produced fewer than 20 colonies counted in the DMSO condition were excluded. For the normal donor CD34+ CFU assays, normal bone marrow was obtained from AllCells. The Human CD34 MicroBead Kit (Miltenyi Biotec, Gaithersburg, MD, USA) was used to isolate CD34+ cells. A total of 1000 CD34+ cells/well were plated with DMSO or 1 µM EPZ in MethoCult H4035 Optimum Without EPO and incubated at 37 °C with 5% CO_2_ and 1% O_2_ for 7–10 days. Colonies were counted by two counters blinded to the experimental conditions using the Revolve Microscope (ECHO). Values are reported as averages of the two separate counters.

### 2.8. MOLM-13 Luciferase Xenograft Murine Model

All animal experiments were conducted after approval of the University of Cincinnati (UC) Institutional Animal Care and Use Committee (IACUC). The 1 × 10^4^ MOLM-13 cells that express luciferase [21] were injected into 8-week-old male NCG mice (Charles River Laboratories, Wilmington, MA, USA) via the tail vein. On day 3 post-engraftment, mice were randomized to receive either vehicle (0.5% methylcellulose, 0.1% Tween80, 99.4% deionized water) or 82 mg/kg EPZ daily oral gavage. On day 10 post-treatment, mice in the EPZ cohort had their dose increased to 300 mg/kg [19,22]. Early removal criteria (ERC) were defined as 20% weight loss, partial or full hindlimb paralysis, dehydration, anorexia, anemia, hunched posture, inactivity, lethargy, difficulty breathing, or rough hair coat.

### 2.9. Primary AML Differentiation

Primary AML cells were cultured for up to 14 days as described above. On day 14, cells were immobilized on glass slides and subsequently stained with Wright-Giemsa stain (Thermo Fisher Scientific). Images were taken on the Revolve Microscope.

### 2.10. Patient-Derived Xenograft (PDX) Murine Studies

Three human AML samples (previously expanded in immunocompromised mice) were treated ex vivo for 14–21 days with DMSO or 1 µM EPZ as described above. We histologically confirmed differentiation in two of the three samples and proceeded with engraftment into immunocompromised NOD.Cg-Rag1tm1Mom Il2rgtm1Wjl Tg(CMV-IL3,CSF2,KITLG)1Eav/J mice (referred to as NRGS mice) [23]. 1 × 10^6^, 2 × 10^5^, 1 × 10^5^, 4 × 10^4^, 2 × 10^4^, or 4 × 10^3^ cells were engrafted into 3–6-month-old NRGS mice via tail vein. A total of 66 male and 63 female recipient mice were used and equally distributed across groups. Twenty-two days post-engraftment, bone marrow aspirates were performed to check for human CD45 (clone H130), human CD33 (clone WM53), and mouse CD45 (clone 30-F11) via flow cytometry (BD FACSCanto II). Mice were monitored for survival until they reached humane endpoints as previously described. Mice that did not reach ERC by 100 days were euthanized to establish if there was any measurable disease. The PDX studies were performed at Cincinnati Children’s Hospital Medical Center under an approved IACUC protocol.

### 2.11. Statistical Analysis

We fitted the ANOVA model for normalized counts about the group for each non-survival dataset, and the parameter estimates and *p*-values are presented [24]. For survival data where some animals remained alive at the study endpoint, we fitted the COX Proportional Hazard model for the survival time of the group using a contrast statement to examine the significance between any two groups [25]. For survival studies where all subjects died by study endpoint, we fitted the ANOVA model for the logarithm of the survival time for the group using a contrast statement. The parameter estimates and *p*-values are provided. All calculations were performed with SAS statistical software (SAS, 2003 Release 9.1 for windows) [26].

## 3. Results

### 3.1. EPZ011989 Is an Effective Inhibitor of EZH2 Function in AML Cell Lines In Vitro and In Vivo

**Figure 1:**
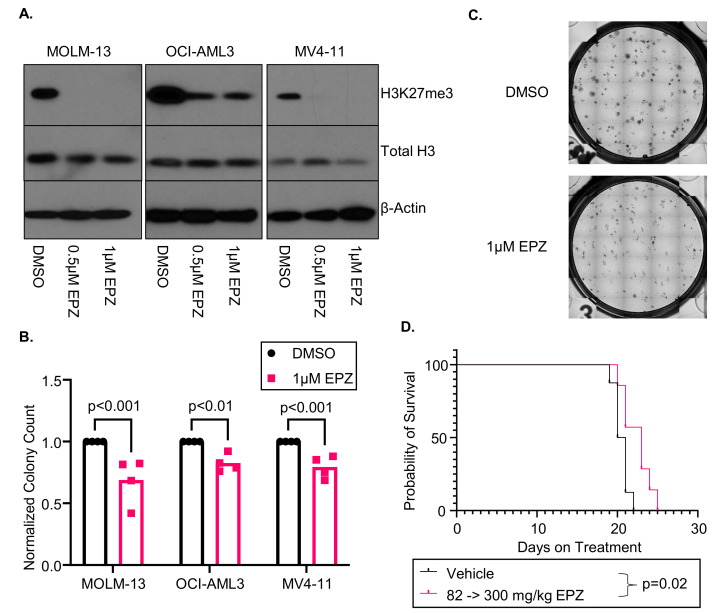
EPZ011989 (EPZ) inhibits H3K27me3 and prevents colony formation in the MOLM-13 cell lines. (**A**) Immunoblot probing for H3K27me3, total H3, and β-actin in MOLM-13, OCI-AML3, and MV4-11 cells after 96 h of treatment with 1 µM of EPZ. Representative image from three replicates. (**B**) Normalized colony counts from a 7-day colony formation assay of MOLM-13, OCI-AML3, and MV4-11 cells treated with 1 µM EPZ (n = 4). (**C**) Representative images from the MOLM-13 colony formation assays shown in 1C. (**D**) Overall survival (in days) of NCG mice engrafted with MOLM-13 luciferase cells and treated with vehicle or 82 mg/kg EPZ and increasing dose to 300 mg/kg EPZ during the study. The uncropped blots are shown in Supplementary File S1.

We first sought to determine if increasing the doses of EPZ011989 (EPZ) reduces H3K27me3 in multiple AML cell lines (MOLM-13, OCI-AML3, and MV4-11). After 96 h of continuous 0.5 or 1 µM EPZ treatment, we found that EPZ decreased H3K27me3 in all three AML cell lines (Figure 1A). However, EPZ only modestly affected the metabolic activity in the MOLM-13 (IC50 = 4.4 µM), OCI-AML3 (IC50 = not reached), and MV4-11 cells (IC50 = not reached). We next investigated the colony forming capabilities in a CFU assay. We found that all cell lines treated for 7 days with 1 µM of EPZ had fewer colonies than the DMSO control (MOLM-13: *p* = 0.001, OCI-AML3: *p* = 0.006, MV4-11: *p* < 0.001; Figure 1B). Furthermore, representative images of the MOLM-13 CFU assay show a striking reduction in the size of the colonies (Figure 1C).

As the MOLM-13 cells had the best response to EZH2 inhibition in vitro, we next wanted to see if the EPZ treatment correlated with improved survival in vivo. The NCG mice were engrafted with MOLM-13 luciferase cells and started treatment 3 days post-engraftment. Disease development was monitored through bioluminescent imaging (Supplemental Figure S1A,B). After 10 days of treatment, we did not see any difference in the disease burden in the 82 mg/kg group, and we increased the dose to 300 mg/kg daily [19,22]. The EPZ-treated mice had a slower development of leukemia, which correlated with a modest improvement in overall survival (20.5 vs. 23 days, *p* = 0.02; Figure 1D). We did not find differences in the weights or complete blood cell counts at ERC between the two cohorts, suggesting that there is no obvious cytopenia or toxicities associated with EPZ treatment (Supplemental Figure S1C–F). This murine model demonstrates that, while EZH2 inhibition is not associated with a dramatic improvement in survival, it reduces the disease burden, suggesting a stronger impact on differentiation rather than cell death. However, cell line models are not optimal systems to investigate AML differentiation due to their immortalized states, and we therefore expanded our studies to primary AML patient samples.

### 3.2. EZH2 Inhibition Induces Differentiation in Primary AML Samples

**Figure 2:**
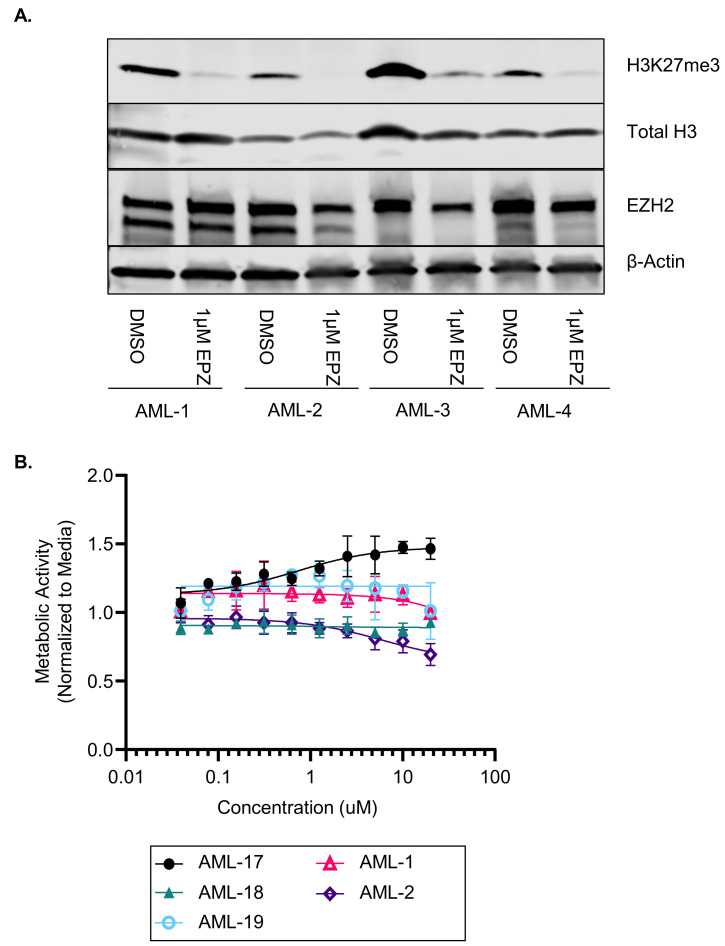
EPZ011989 induces differentiation in primary AML samples in vitro. (**A**) Representative immunoblot probing for H3K27me3, total H3, EZH2, and β-actin after 7 days of treatment with DMSO or 1 µM of EPZ (total n = 8). (**B**) Metabolic activity assay from primary AML samples treated with increasing concentration of EPZ for 7 days (n = 5). The uncropped blots are shown in Supplementary File S2.

**Figure 3:**
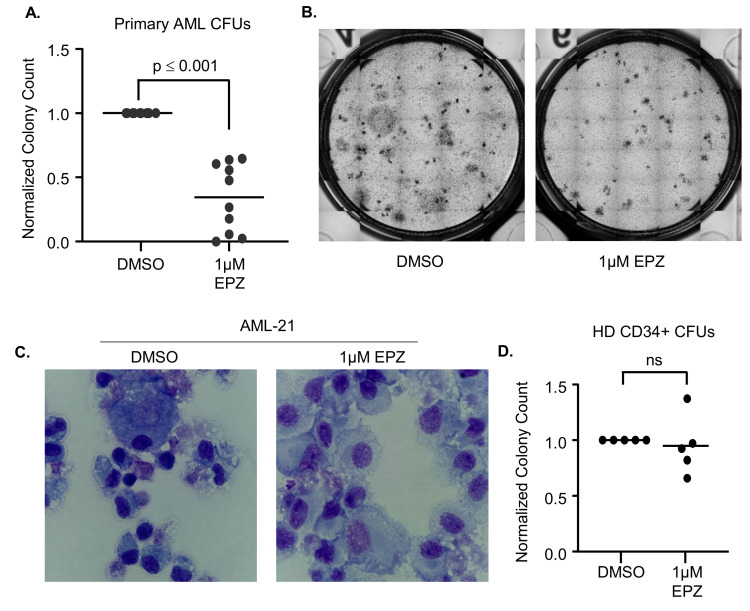
EZH2 inhibition reduces colony formation in primary AML samples but not in normal donor CD34+ cells. (**A**) Normalized colony counts from CFU assays where primary AML samples were treated with DMSO or 1 µM of EPZ for 14 days (n = 6). (**B**) Representative images from the primary AML samples’ CFU assays shown in Figure 3A. (**C**) Representative images of primary AML cells after 14 days of in vitro treatment with DMSO or 1 µM of EPZ. (**D**) Normalized colony counts from CFU assays of CD34+ normal donor cells treated with DMSO or 1 µM of EPZ for 14 days (n = 5); ns—not significant.

Given the limitations of studying differentiation and stemness in a homogeneous cell line model, we next performed studies with H3K27me3 inhibition in primary AML samples (characteristics of all primary samples are provided in Supplemental Table S1). We first confirmed that EPZ could reduce the H3K27me3 levels in primary AML samples after 7 days of treatment (Figure 2A, N = 4 of 8 samples treated). However, this inhibition did not impact the metabolic activity of primary AML cells via MTS assays after 7 days of EPZ treatment (n = 5, Figure 2B). However, we noted a modest increase in differentiation markers (CD11b, CD14, CD16, and CD38) on the cell surfaces of the primary AML samples after 7 days of EZH2 inhibition (Supplemental Figure S2). Differentiation therapy has been successful in AML because differentiated cells have decreased stemness and are more susceptible to natural cell turnover and death; however, this is the most evident after prolonged exposure to the drug. Thus, we performed 14-day CFU assays with primary AML samples continuously treated with DMSO or 1 µM of EPZ. We found a significant reduction in the total number of colonies formed (*p* < 0.001, Figure 3A) as well as a reduction in the colony size (Figure 3B). In addition, 14 days of 1 µM of EPZ treatment in a liquid culture resulted in morphological differentiation, as seen through the increased granularity in the cytoplasm and the decreased nuclear size (Figure 3C, Supplemental Figure S3). Interestingly, in the healthy donor bone marrow, we did not see the same reduction in colony number (*p* = 0.67, Figure 3D). These data suggest that there is a therapeutic window for targeting EZH2 in AML cells without impacting normal hematopoiesis.

### 3.3. Inhibition of EZH2 Prior to Engraftment Delays Expansion of Primary AML Cells

**Figure 4:**
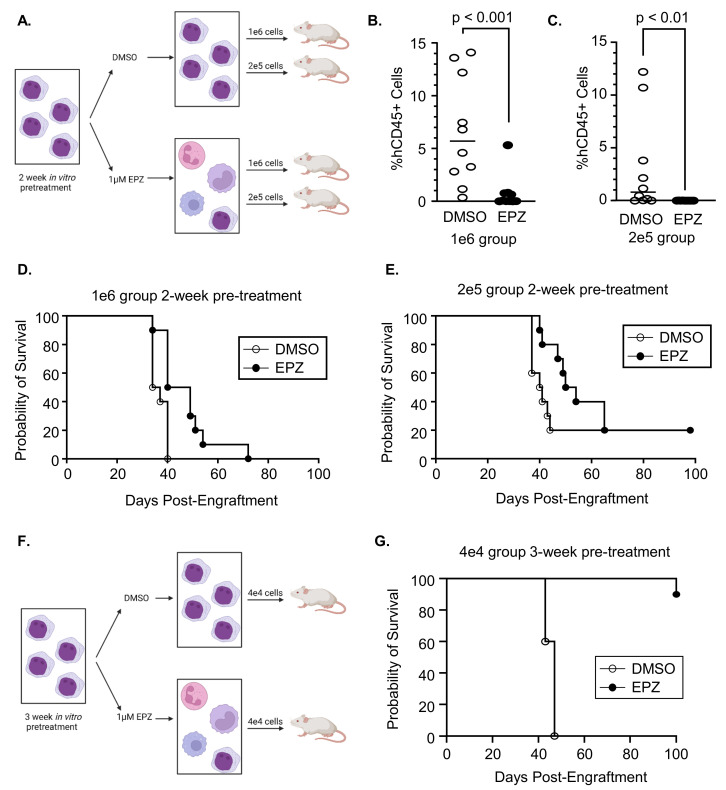
Pretreatment of AML blasts with EPZ delays engraftment into NRGS mice. (**A**) Schematic of experimental design for 2 weeks of pretreatment. Percent of human CD45 (hCD45) detected in bone marrow aspirates of mice engrafted with (**B**) 1 × 10^6^ or (**C**) 2 × 10^5^ DMSO cells or cells pretreated with 1 µM of EPZ for 2 weeks on day 22 of engraftment (n = 10 mice per group). Overall survival of mice engrafted with (**D**) 1 × 10^6^ or (**E**) 2 × 10^5^ DMSO or AML-8 cells pretreated with 1 µM of EPZ for 2 weeks (n = 10 mice per group). (**F**) Schematic of experimental design for 3 weeks of pretreatment. (**G**) Overall survival of mice engrafted with 4 × 10^4^ DMSO or AML-8 cells pretreated with 1 µM of EPZ for 3 weeks (n = 10 mice per group).

As EZH2 inhibition induces the differentiation of primary AML samples, we hypothesized that treatment could impact stemness and therefore delay engraftment into NRGS mice. To test this hypothesis, we used human AML samples that had been previously expanded in an immunodeficient mouse and treated them with DMSO or 1 µM of EPZ for 2 weeks ex vivo. We screened three samples and confirmed our previous findings that EPZ inhibits H3K27me3 and induces differentiation (Supplemental Figure S4A). We selected sample AML-8 and engrafted 1 × 10^6^ or 2 × 10^5^ cells into NRGS mice after 2 weeks of pretreatment with DMSO or 1 µM of EPZ (Figure 4A). After 22 days of engraftment, bone marrow aspirates were performed to assess the disease burden. The mice engrafted with EPZ-treated cells had a significantly lower disease burden compared to the DMSO-treated cells regardless of the number of cells engrafted (Figure 4B,C; 1 × 10^6^ engrafted cells: *p* < 0.001; 2 × 10^5^ engrafted cells: *p* = 0.002). Furthermore, engrafting mice with EPZ-treated cells prolonged survival at both cell doses compared to the mice engrafted with DMSO-treated cells (Figure 4D,E; median survival of 1 × 10^6^ DMSO- vs. EPZ-treated cells: 35.5 vs. 44.5 days, *p* = 0.04; median survival of 2 × 10^5^ DMSO- vs. EPZ-treated cells: 40.5 vs. 52 days, *p* = 0.07). We confirmed these results using a second AML donor (AML-7) treated for 2 weeks prior to engraftment and saw a similar trend at higher cell doses (Supplemental Figure S4B).

Finally, we pretreated AML-8 cells with DMSO or 1 µM of EPZ for 3 weeks prior to engrafting 4 × 10^4^ cells (versus 2 weeks, as described above) to determine if a longer treatment further impacted engraftment and survival (Figure 4F). All mice engrafted with DMSO-treated cells met ERC by day 47, while only one of the mice engrafted with EPZ-pretreated cells met ERC after 100 days of engraftment (Figure 4G, *p* < 0.001). Of the remaining nine mice engrafted with EPZ-pretreated cells at day 100 post-engraftment, three mice had measurable disease (98%, 28%, and 0.3% human CD45). These results suggest that the inhibition of EZH2 promotes the differentiation of AML blasts and subsequently delays engraftment and improves survival in vivo.

## 4. Discussion

The role of EZH2 in AML can be contradictory, as it can function as a tumor suppressor prior to AML development and an oncogene after AML development [18]. Therefore, it is important to contextualize the results of studies with EZH2 inhibitors based on whether the AML is in the development or maintenance phase. While we were able to show that EPZ did not reduce colony formation in normal donor CD34+ cells, we have not interrogated the effect of EZH2 inhibition on other hematopoietic cells. Previous studies in lymphoma suggest that EZH2 inhibition can impact B cells regardless of the EZH2 mutational status. Furthermore, the loss of EZH2 in T cells is associated with decreased anti-tumor immune surveillance [27]. While the relative contribution of B cells and T cells in advanced AML is low, the impact of EZH2 inhibition on the tumor microenvironment should be carefully considered.

In addition, the complex genetic background in AML is a significant factor impacting the response to therapy. A recent study found that EZH2 inhibition in FLT3-mutant AML further enhanced the differentiation effect [28]. Similarly, another study found that the inhibition of the MEK/ERK and PI3K/AKT pathways reduce the total EZH2 and H3K27me3 levels, resulting in a reduced transformation to AML [29]. Nevertheless, our data provided herein support the use of EZH2 inhibitors only in patients with established AML disease, regardless of FLT3 or other mutations. This is even the case in patients with established EZH2-inactivating mutations. While none of the samples in our current series included EZH2 mutations, these patients might also benefit from EZH2 inhibition based on a recent study that describes that cell death pathways are upregulated in AML patient cells with mutant EZH2 [30].

We have shown that EPZ011989 can induce differentiation in AML cell lines and primary AML samples. EPZ treatment reduces the colony formation capabilities of MOLM-13, OCI-AML3, and MV4-11 cells in CFU assays, which correlates with an improved survival and a reduced disease burden in a MOLM-13 luciferase xenograft model. While EZH2 inhibition is not cytotoxic to primary AML cells in vitro, it induces differentiation, as seen by histologic changes and reduced colony formation capabilities. This induction of differentiation results in a delayed engraftment into NRGS mice after 2 or 3 weeks of pretreatment with an EZH2 inhibitor in a cell in a dose-dependent manner. A previous study similarly found that EPZ011989 (prepared in a nanoformulation) has a similar efficacious role in AML; however, this study focused on the mechanistic impact of proteasomal degradation rather than the differentiation aspect [31].

There is still more to uncover regarding how EZH2 functions in AML and which specific genes are altered by EZH2 dysregulation. Some studies suggest that EZH2 mutations or loss of function is a poor prognostic factor in AML and promote resistance to therapy [16,17]. Our studies show that EZH2 inhibition in AML has little cytotoxic impact but effectively induces differentiation. In the PDX studies where cells were treated for 2 weeks prior to transplant into recipient mice, some mice in the EPZ-treated arms still met ERC, suggesting that either 1 µM of EPZ was not sufficient to induce terminal differentiation in all cells or that the treatment did not induce a durable differentiated state. Future studies should interrogate potential combination therapies to overcome these limitations. Combination therapy with EZH2 inhibition might be able to reduce the disease burden so that more definitive treatments could be used, such as stem cell transplant. Porazzi et al. recently published that EZH2 inhibition, in combination with cytotoxic agents, enhanced the cell killing of AML cells, which would potentially allow for lower chemotherapeutic doses to be used [32]. In addition, as EZH2 inhibition increases cell surface markers associated with differentiation, such as CD38, there is a space to study combination therapies with immunotherapies and monoclonal antibodies (i.e., daratumumab). Combination approaches with other histone-modifying agents is also a possibility, as shown with the EZH2 inhibitor EPZ6438 (tazemetostat) combined with an LSD1 inhibitor [33]. A recent study using a multi-omics approach identified a transcriptional network driven by both gain-of-function and loss-of-function mutations in EZH2, including the identification of potential targets that could provide better combination approaches with EZH2 inhibitors [34]. These combination strategies would provide the potential to induce a more durable remission with fewer treatment side effects. Additional studies like these will be needed to rationally design successful therapeutic combinations. We found one study actively recruiting for the combination of the EZH2 inhibitor tazemetostat in combination with CPX-351 in relapsed and refractory AML (NCT05627232). There is still much research needed to undercover EZH2’s complex role in AML; nevertheless, our data support the additional clinical translation of these inhibitors as single agents or in combination.

## 5. Conclusions

In summary, we have shown that EPZ011989 can inhibit the function of EZH2, as evidenced by reduced levels of H3K27me3, and promote the differentiation of AML cells. The differentiation of AML blasts due to EZH2 inhibition prior to engraftment prolongs survival in NRGS mice. Our study demonstrates the importance of understanding the mechanism of EZH2 in AML to develop effective future therapeutics in AML.

## Data Availability

All data are contained within the article or Supplementary Materials or are available from the corresponding author upon request.

## Supplementary Materials

The following supporting information can be downloaded at https://www.mdpi.com/article/10.3390/cancers16030569/s1, Table S1: AML primary patient sample characteristics. Figure S1: EPZ-treated mice did not have any therapy-related anemia or thrombocytopenia. Figure S2. EPZ011989 induces surface markers of differentiation in primary AML samples. Figure S3. EZH2 inhibition induces phenotypic differentiation in primary AML cells. Figure S4. EZH2 inhibition in primary AML samples induces differentiation, which delays engraftment into NRGS mice. Files S1 and S2. Quantification of immunoblots. Additional supplemental data.
