# Supplementary material for: Inhibition of Enhancer of Zeste Homolog 2 Induces Blast Differentiation, Impairs Engraftment and Prolongs Survival in Murine Models of Acute Myeloid Leukemia
Source: Cancers (Basel). 2024 Jan 29;16(3):569. doi: 10.3390/cancers16030569 (PMC10854504; doi:10.3390/cancers16030569)

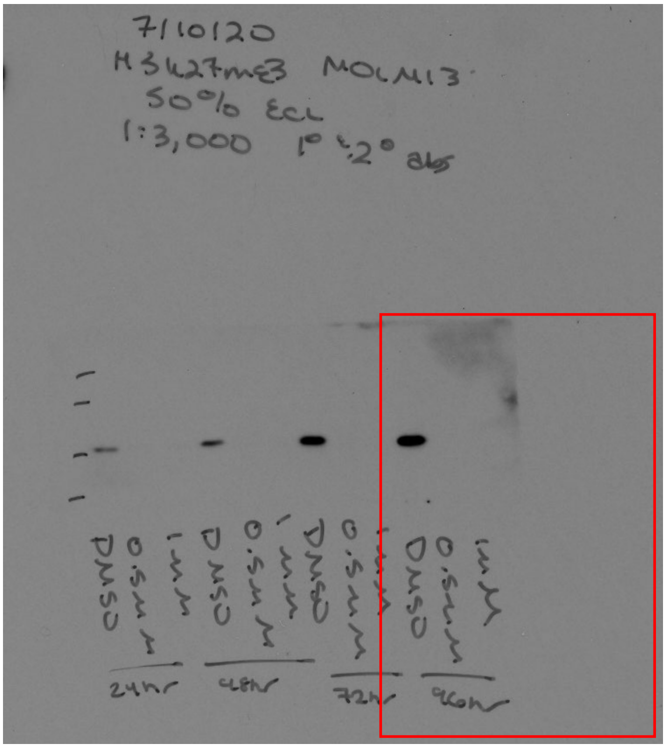

7/10/20
H3K27me3 MOLM13
50% ECL
1:3,000 1° & 2° abs
24hr
48hr
72hr
96hr
MOLM-13
H3K27me3
DMSO
0.5μM EPZ
1μM EPZ

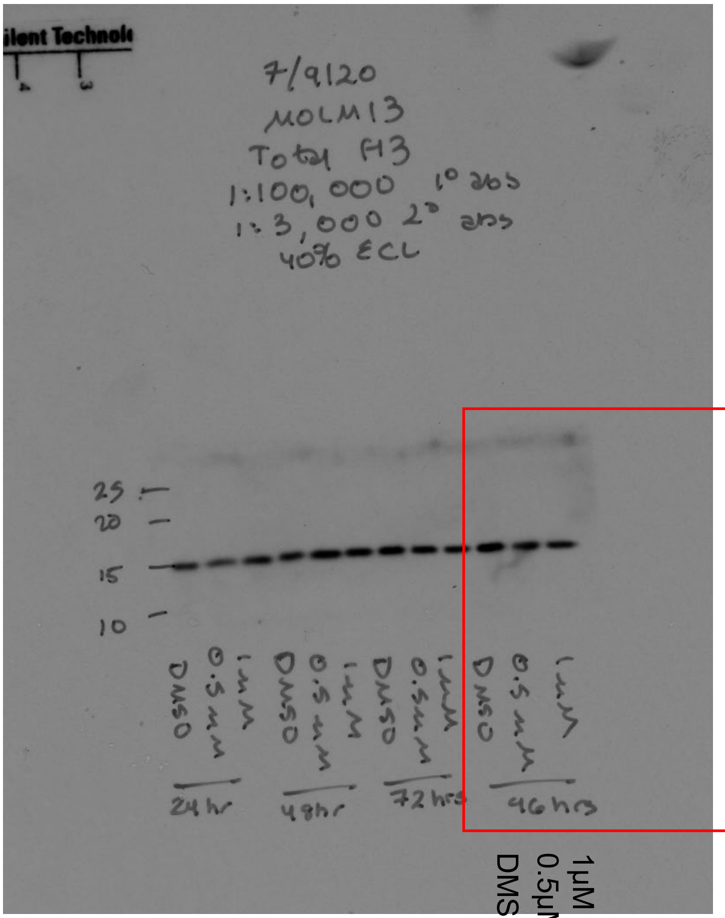

7/9/20
MOLM13
Total H3
1:100,000 1° abs
1:3,000 2° abs
40% ECL
25
20
15
10
24 hr
48hr
72 hrs
96 hrs
MOLM-13
Total H3
DMSO
0.5μM EPZ
1μM EPZ

A.

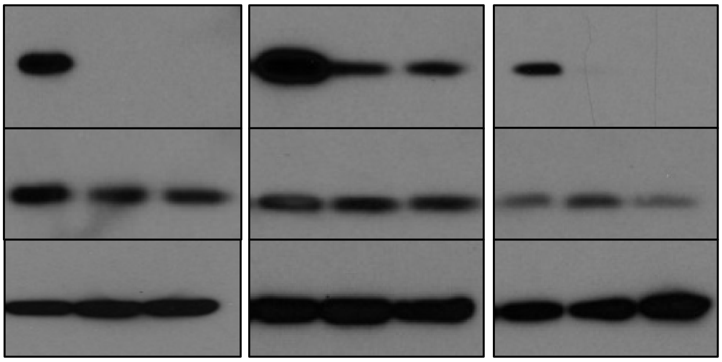

MOLM-13
OCI-AML3
MV4-11
H3K27me3
Total H3
β-Actin
DMSO
0.5μM EPZ
1μM EPZ
DMSO
0.5μM EPZ
1μM EPZ
DMSO
0.5μM EPZ
1μM EPZ

MOLM-13

β–Actin

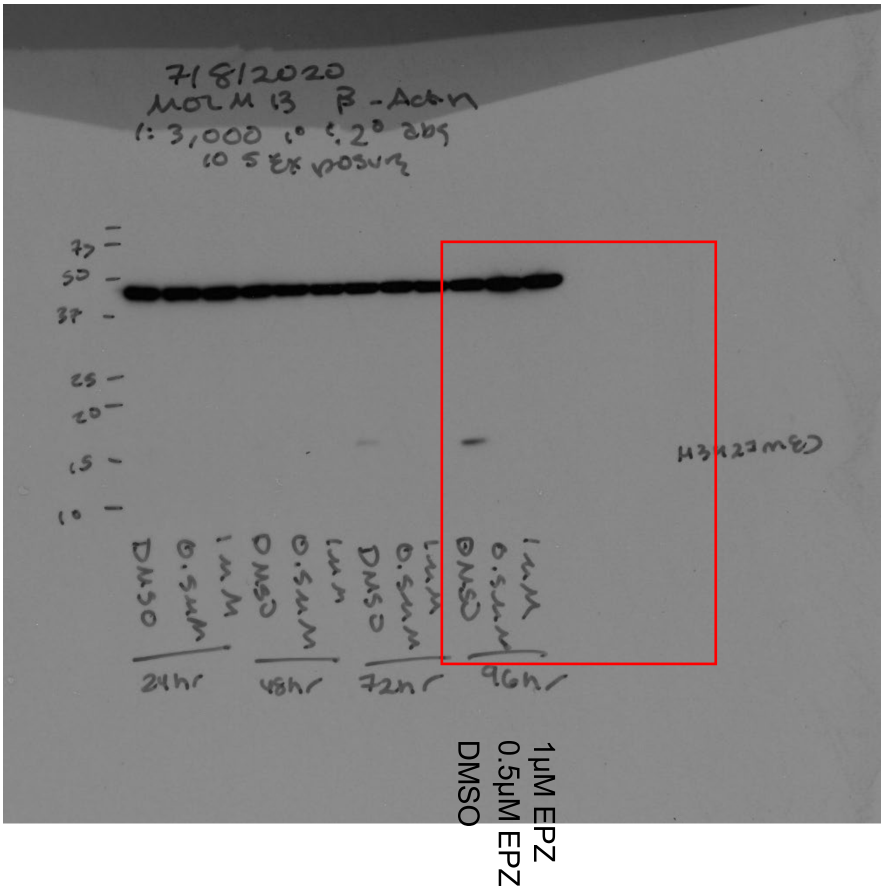

A.

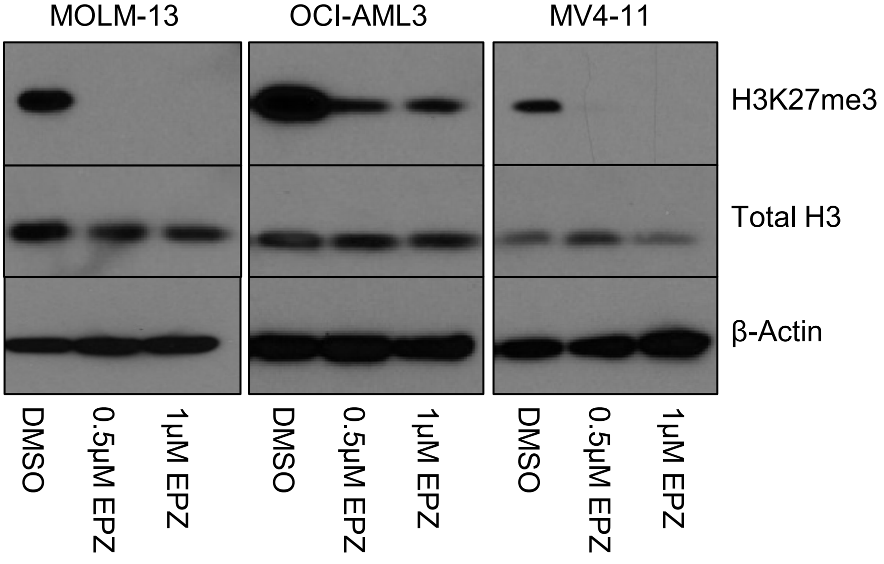

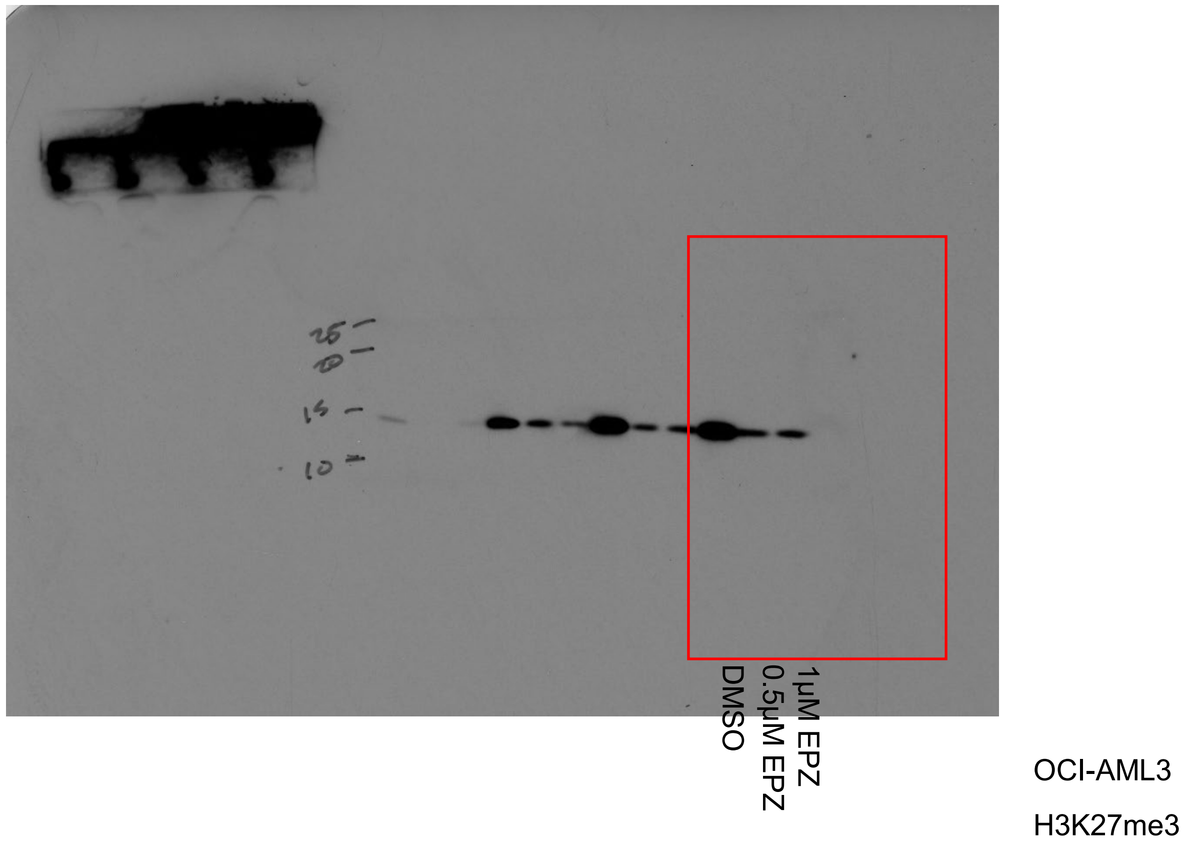
DMSO
0.5μM EPZ
1μM EPZ
OCI-AML3
H3K27me3

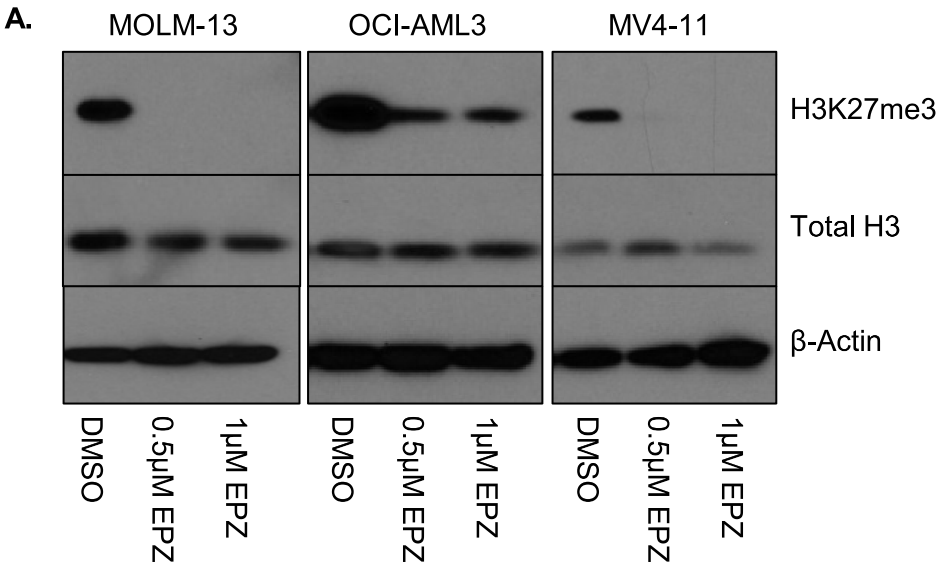
A.
MOLM-13
OCI-AML3
MV4-11
H3K27me3
Total H3
β-Actin
DMSO
0.5μM EPZ
1μM EPZ
DMSO
0.5μM EPZ
1μM EPZ
DMSO
0.5μM EPZ
1μM EPZ

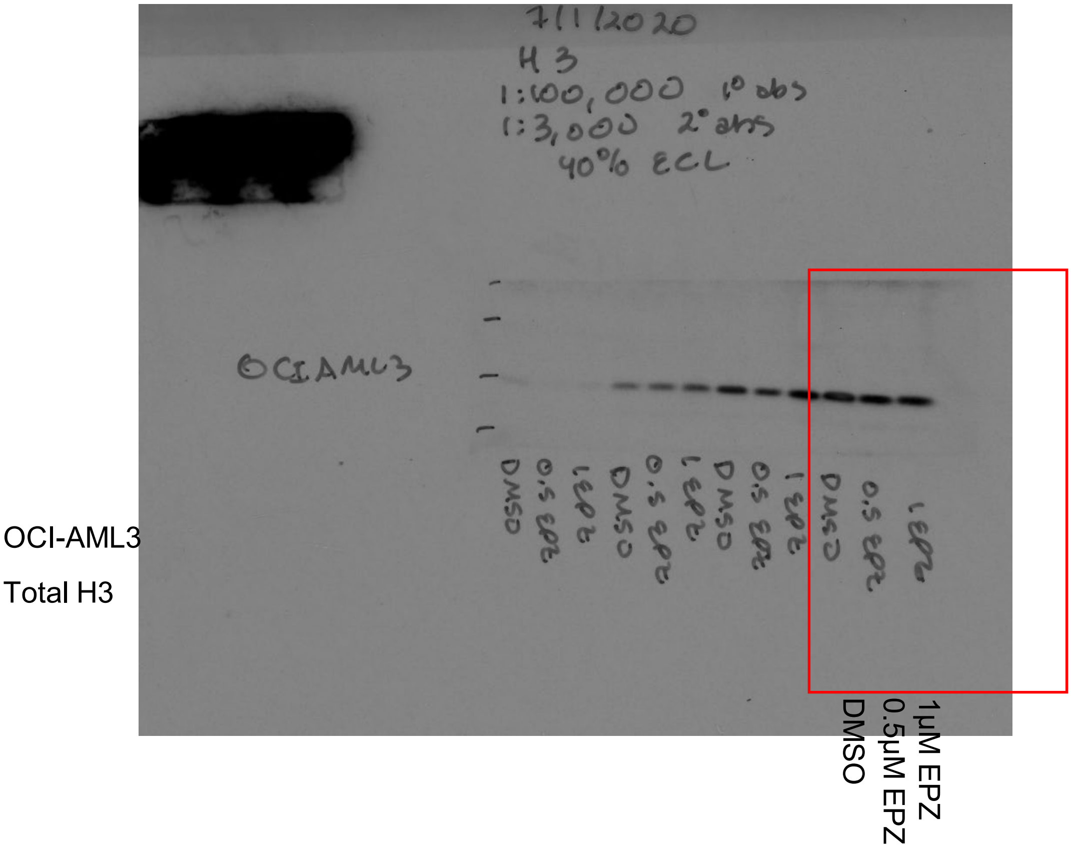
7/1/2020
H3
1:100,000 1° abs
1:3,000 2° abs
40% ECL
OCIAML3
OCI-AML3
Total H3
DMSO
0.5μM EPZ
1μM EPZ

A.

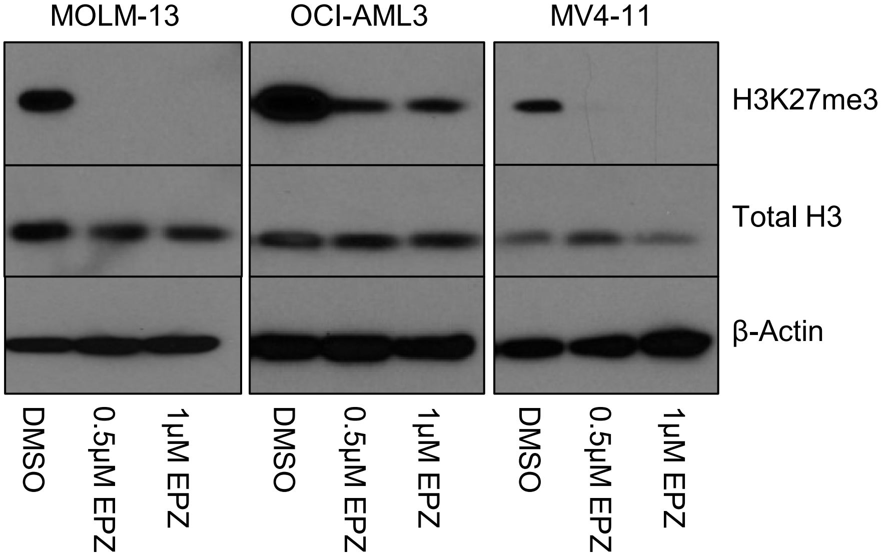
MOLM-13
OCI-AML3
MV4-11
H3K27me3
Total H3
β-Actin
DMSO
0.5μM EPZ
1μM EPZ
DMSO
0.5μM EPZ
1μM EPZ
DMSO
0.5μM EPZ
1μM EPZ

MV4-11

H3K27me3

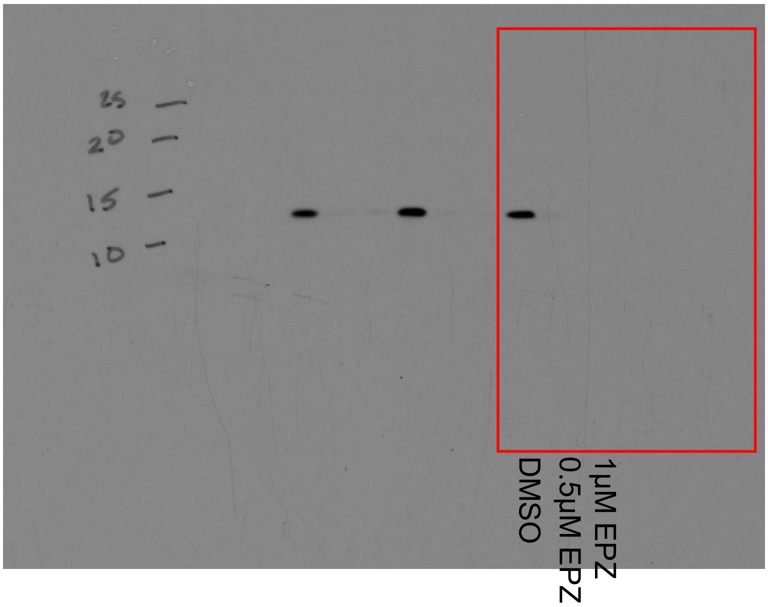

MV4-11

Total H3

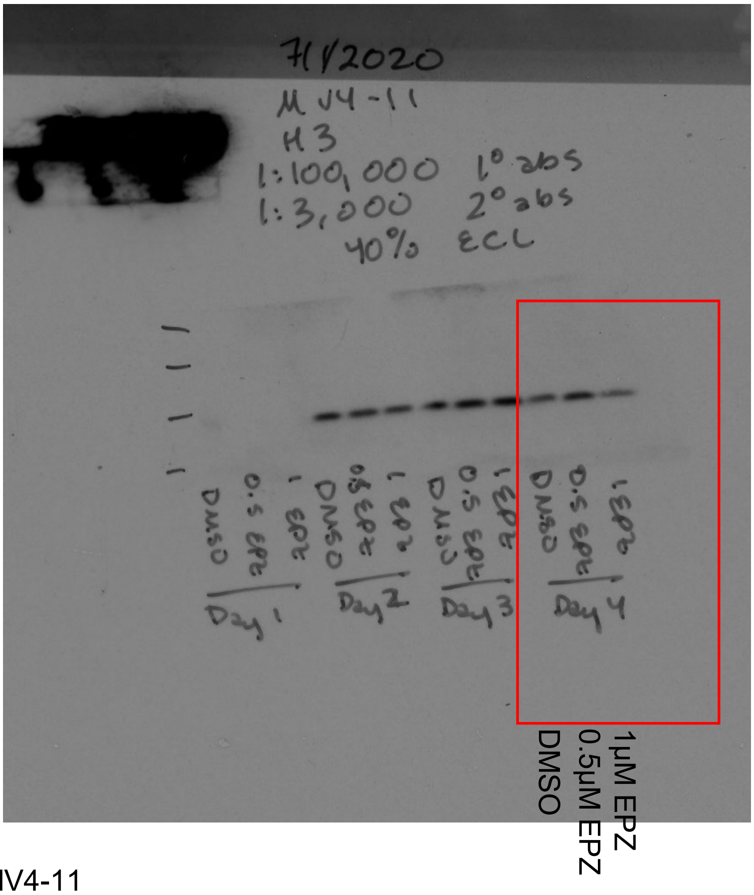

A.

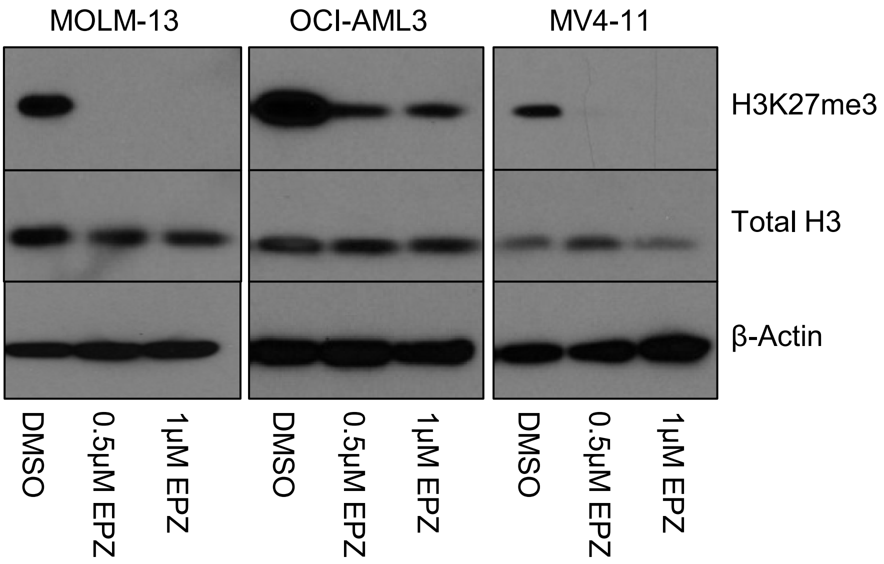

OCI-AML3

β–Actin

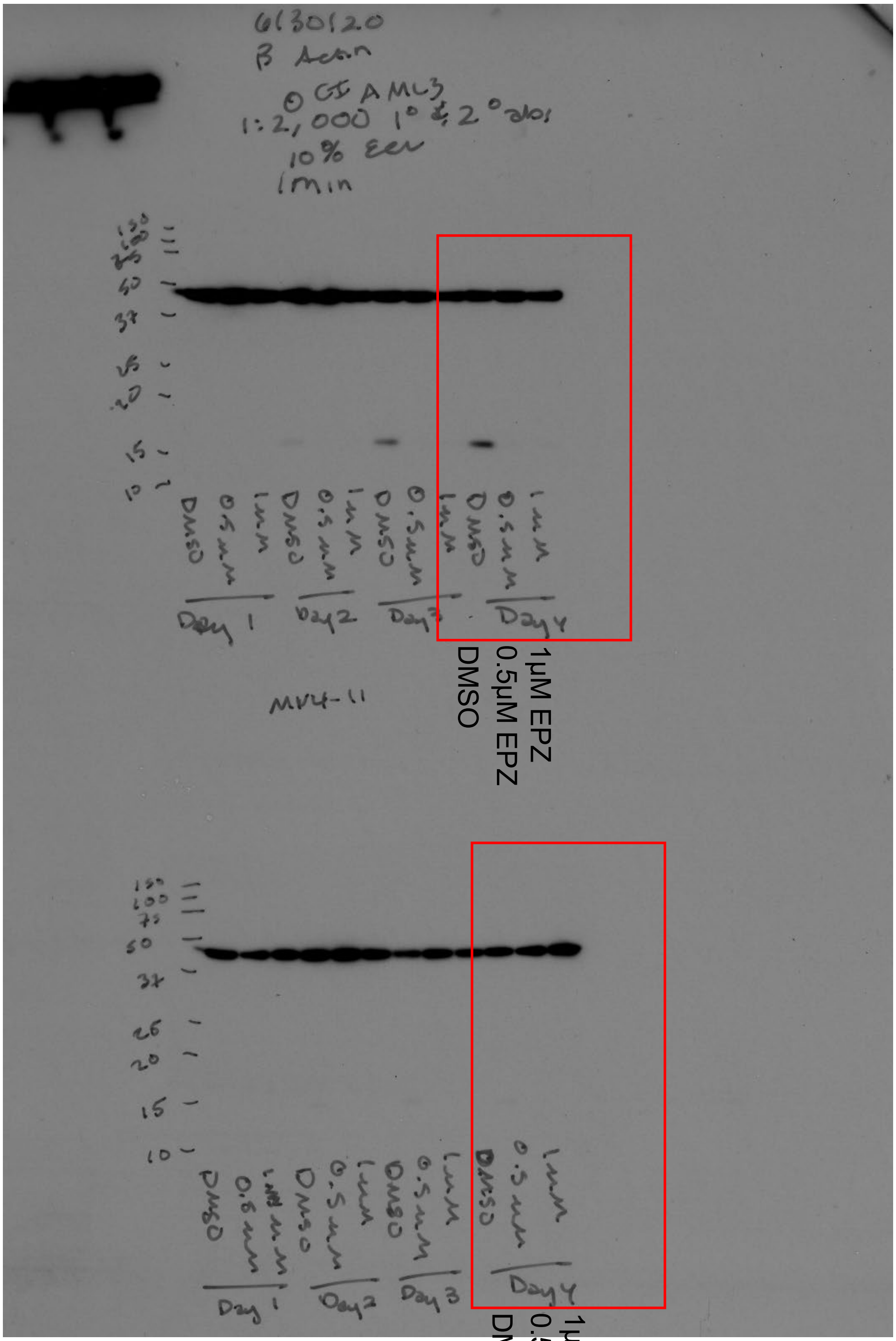

MV4-11

β–Actin

A.

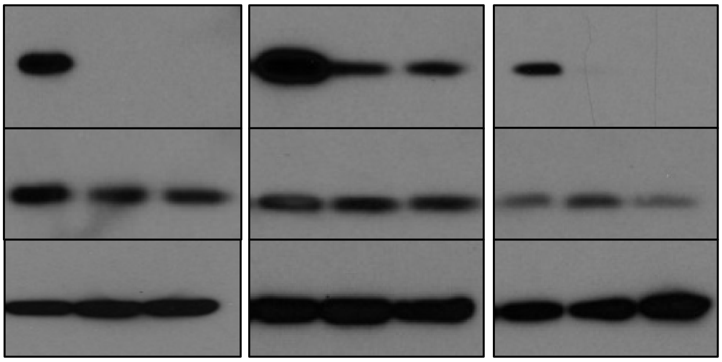

Supplement: Supplementary file 1 [file cancers-16-00569-s001.zip › File S1_uncropped immunoblots for Figure 1.pdf]
