# Supplementary material for: Inhibition of Enhancer of Zeste Homolog 2 Induces Blast Differentiation, Impairs Engraftment and Prolongs Survival in Murine Models of Acute Myeloid Leukemia
Source: Cancers (Basel). 2024 Jan 29;16(3):569. doi: 10.3390/cancers16030569 (PMC10854504; doi:10.3390/cancers16030569)
Supplement: Supplementary file 1 [file cancers-16-00569-s001.zip › Supplemental Table S1 Patient Sample Characteristics.pdf]

Supplemental Table S1: AML Patient Sample Characteristics

Unique ID	Sample source	Genetic mutations	Karyotype
AML-1	Blood	ASXL / DNMT3A	47,XX,+6,dmin[cp20].nuc ish(amp MYC+)
AML-2		IDH2 R140Q	46,XY[20]
AML-3	Apheresis	Philadelphia + / BCR-ABL	48,XX,+8,t(9;22)(q34;q11.2),+der(22)t(9;22)(q34;q11.2)[20]
AML-4	Apheresis	None Detected	46,XY,inv(16)(p13.1q22)[14]/46,XY[5]/nonclonal with clonal abnormalities[1]
AML-5	Blood	TP53 / DNMT3A	45,XX,add(3)(p21),add(4)(q21),del(5)(q15q35),-9,der(11)(pter->q23::q21->q23::q13->qter),del(12)(p11.2),-13,-13,-15,-19,+mar1,+mar2,+mar3,+mar4[13,two w/nonclonal abnormalities]/
AML-6	Apheresis	TET2 T229fs / NPM1 W288fs / FLT3-ITD E596	46,XX[20]
AML-7	Bone marrow	CDKN2A/B loss of p16INK4a and p14ARF exon 1 / loss of CDKN2B	complex karyotype
AML-8	Bone marrow	UNK	UNK
AML-9	Bone marrow	None Detected	46,XX[20]
AML-10	Bone marrow	None Detected	47,XY,t(1;11)(p32;q23),+19[20]
AML-11	Bone marrow	None Detected	46,XY,t(5;20)(q15;q13.1),add(19)(p13.3),add(19)(p13.3)(cp9)/46,idem,del(9)(
AML-12	Apheresis	KMT2A (MLL) rearrangement / FLT3-JMD	46,XY,t(6;11)(q27;q23)[19]/nonclonal[1]
AML-13	Bone marrow	None Detected	46,XY,t(12;22)(p13;q11.2)[6]/46,sl,t(X;13)(q13;q12)[4]/47,sdl1,+8[10].ish t(12;22)(3' ETV6+;5' ETV6+)
AML-14	Apheresis	NPM1 / BCR-ABL	46,XY,t(9;22)(q34;q11.2)[18]/nonclonal w/clonal[2]
AML-15	Bone marrow	FLT3-ITD	46,XX[20]
AML-16	Blood	IDH1 / JAK2	46,XY[20]
AML-17	Apheresis		46,XY,inv(16)(p13q22)[14]/46,XY[4]/nonclonal w/clonal abnormality[2]
AML-18	Apheresis	FLT3-ITD F605 / NPM1 W288fs / NRAS G12D/ RAD21 G547 / WT1 A382 / PTPN11	46,XY[20]
AML-19	Apheresis	NPM1	Insufficient Metaphases (46,XX[1])
AML-20	Bone marrow	NPM1	46,XY,del(7)(p15)[20]
AML-21	Apheresis	FLT3-ITD	46,XY[20]
AML-22	Apheresis	GATA2 / NPM1 / FLT3-ITD	46,XX[19]/nonclonal[1]
AML-23	Blood	IDH1 R132H / NPM1 W288 / PTPN11 N58Y / DNMT3A R882 / PTPN11 S502L	46,XY[19]/nonclonal[1]
AML-24	Apheresis	FLT3-ITD / IDH2 R140Q / DNMT3A R882H	46,XX[19]/nonclonal[1]

Supplemental Table S1: AML Patient Sample Characteristics (Cont'd)

Unique ID	Sample source	Genetic mutations	Karyotype
AML-25	Bone marrow	None Detected	46,XX,t(1;19)(p36.1;p13.3)?c,t(9;22)(q34;q11.2)[20]
AML-26	Apheresis	NPM1 / DNMT3A / IDH2 / NRAS	46,XX[20]
AML-27	Blood		46,XY,t(15;17)(q24;q21)[10]
AML-28	Blood	CEBPA / JAK2 / WT1 R250P / WT C244 / TET2	46,XX[20]
AML-29	Bone marrow	NF1 Q1775* / PTPN11 A461G (subclonal) / CDKN2A/B loss / ETV6 loss / PICALM-MLLT10 fusion / PHF6 R274Q / TP53 K164E (subclonal) and Y126*	complex karyotype
