# Supplementary figures and images for: Inhibition of Enhancer of Zeste Homolog 2 Induces Blast Differentiation, Impairs Engraftment and Prolongs Survival in Murine Models of Acute Myeloid Leukemia
Source: Cancers (Basel). 2024 Jan 29;16(3):569. doi: 10.3390/cancers16030569 (PMC10854504; doi:10.3390/cancers16030569)

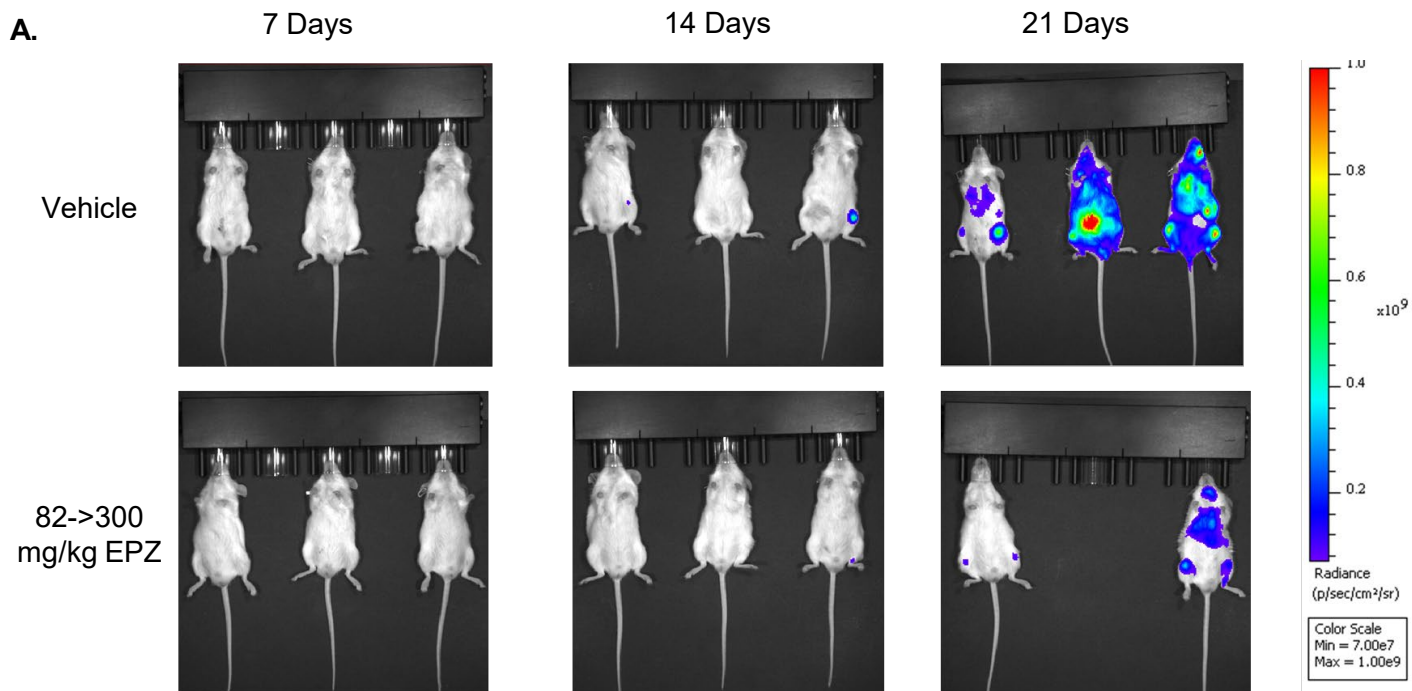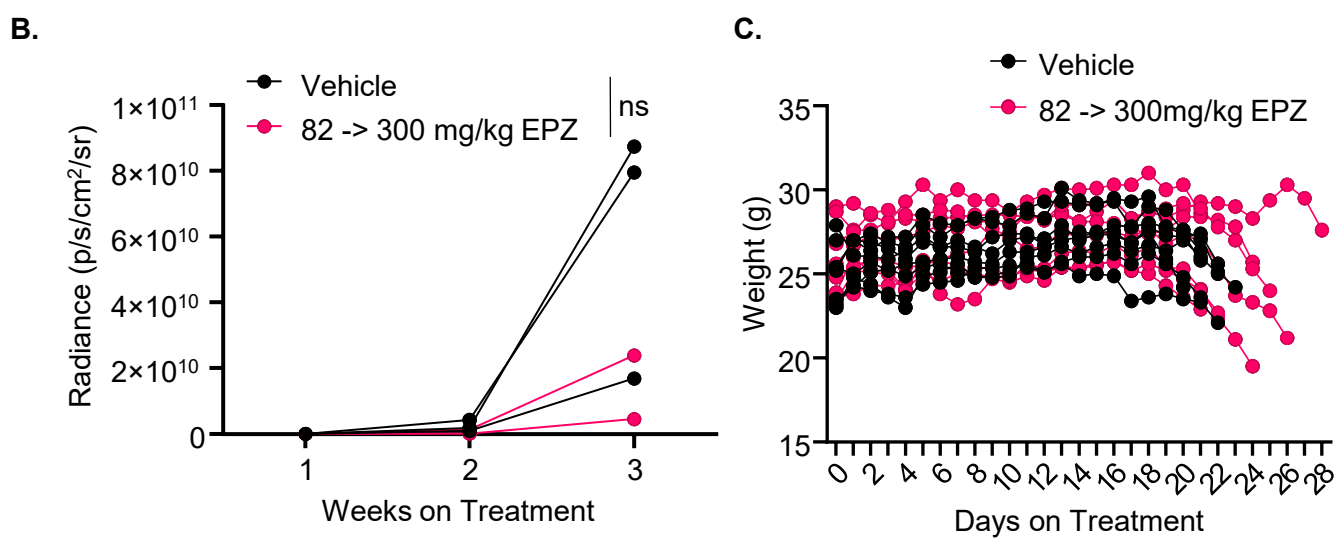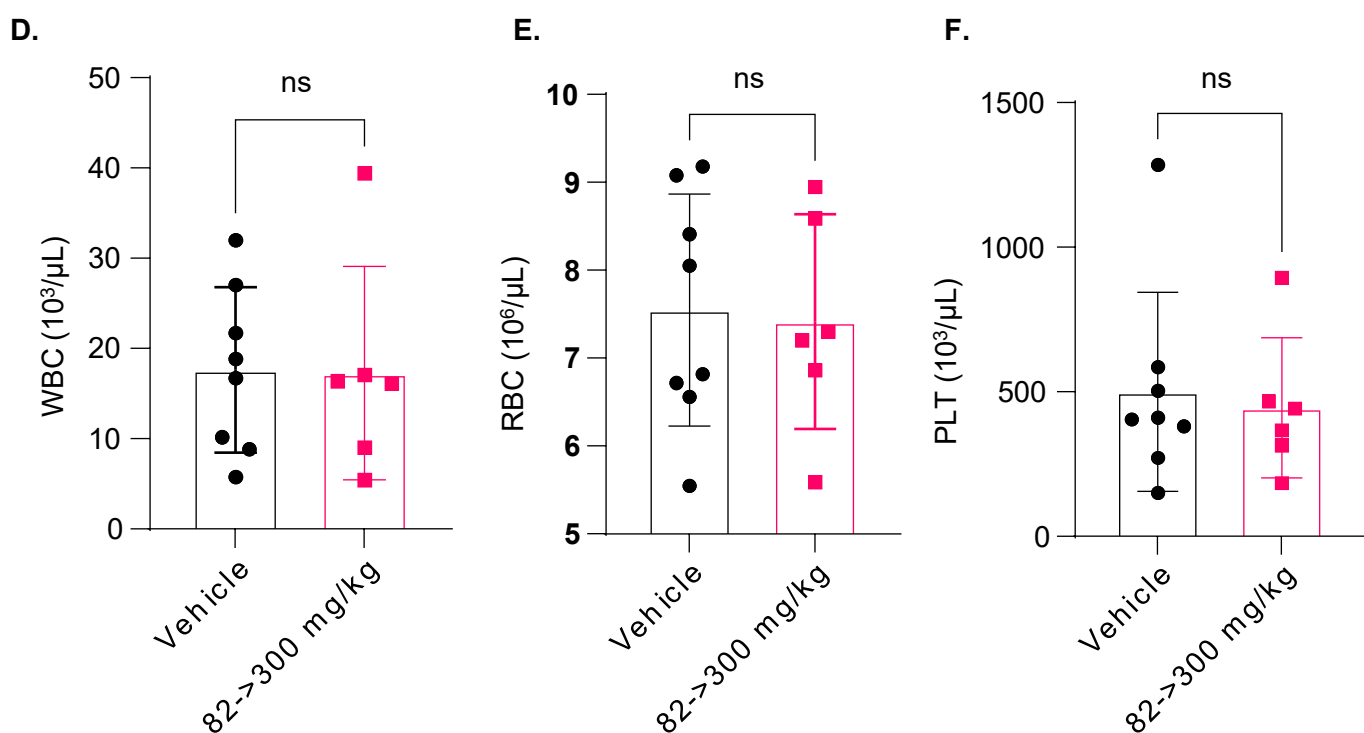

Supplement: Supplementary file 1 [file cancers-16-00569-s001.zip › 20240105 EZH2 Supp Figure S1_Final.pdf]

A.

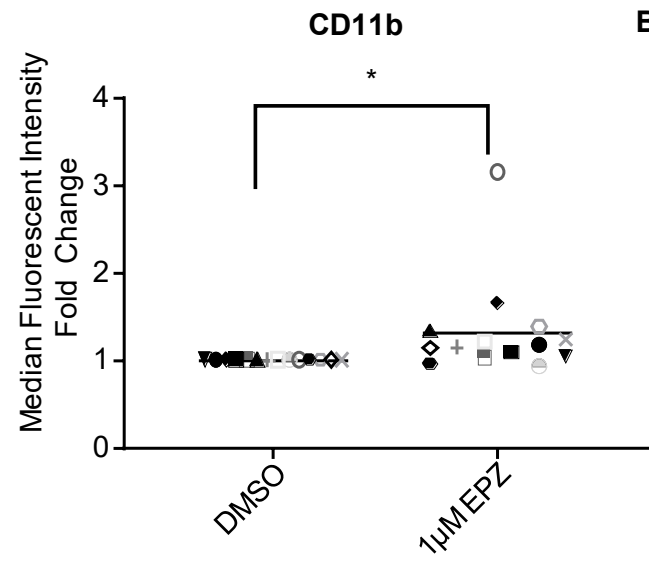

B.

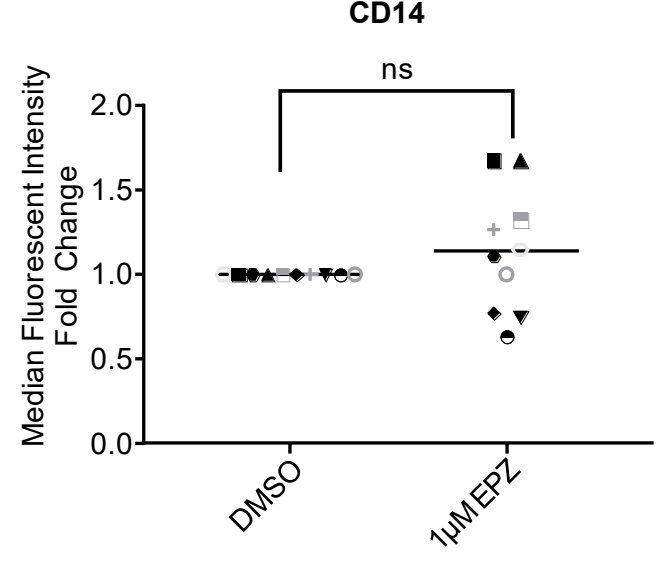

C.

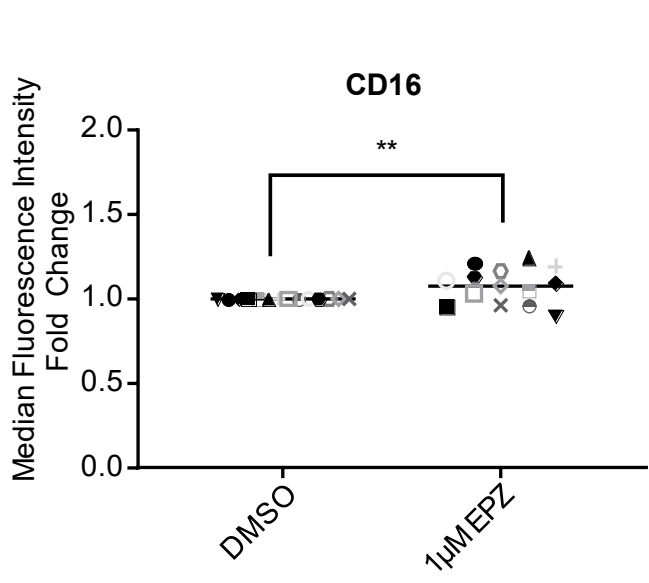

D.

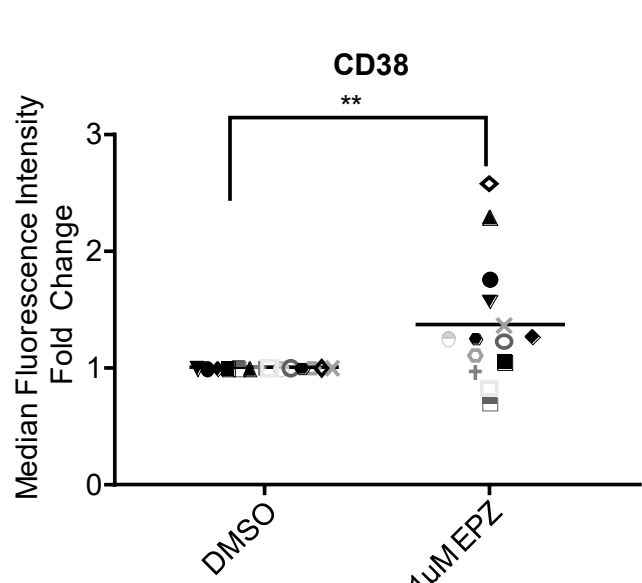

Supplement: Supplementary file 1 [file cancers-16-00569-s001.zip › 20240105 EZH2 Supp Figure S2_Final.pdf]

AML-25

DMSO

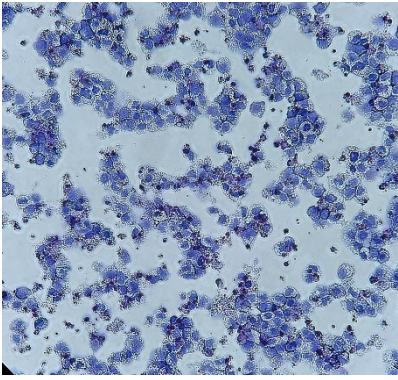

1 $\mu$ M EPZ

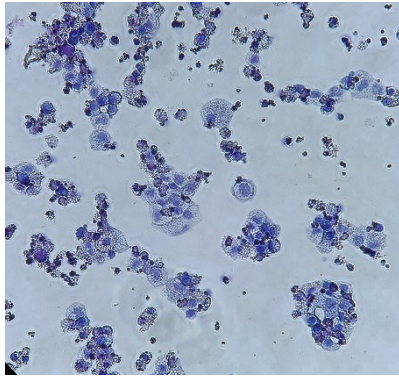

AML-27

DMSO

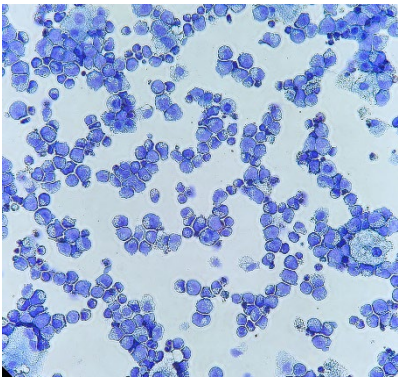

1 $\mu$ M EPZ

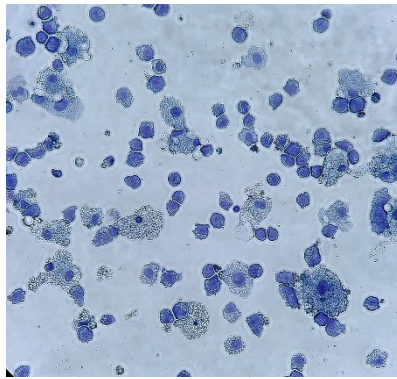

AML-1

DMSO

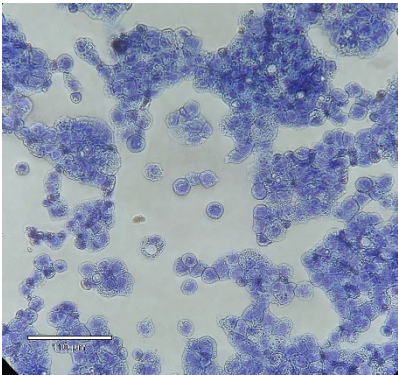

1 $\mu$ M EPZ

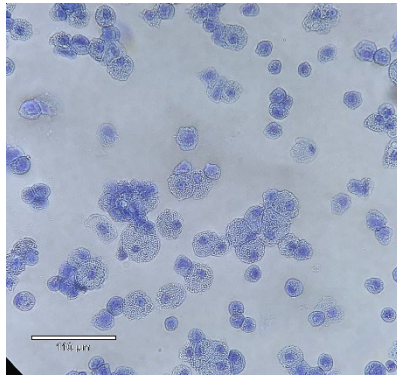

Supplement: Supplementary file 1 [file cancers-16-00569-s001.zip › 20240105 EZH2 Supp Figure S3_Final.pdf]

**A.**

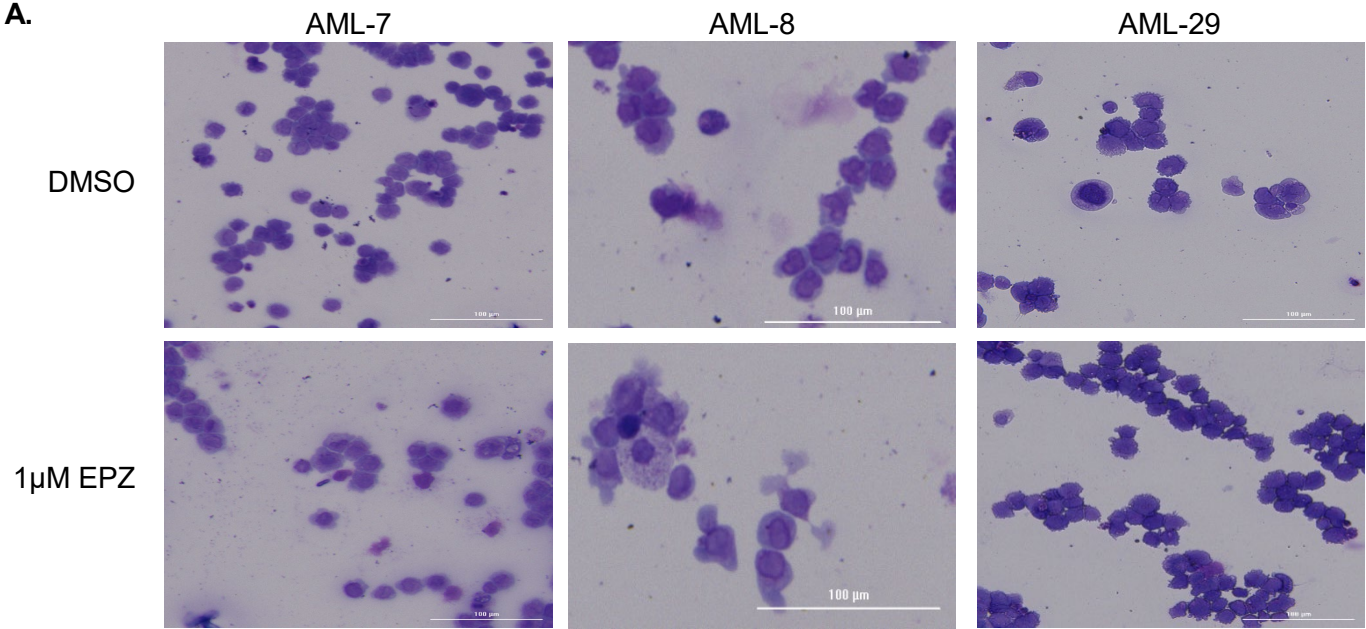

**B.**

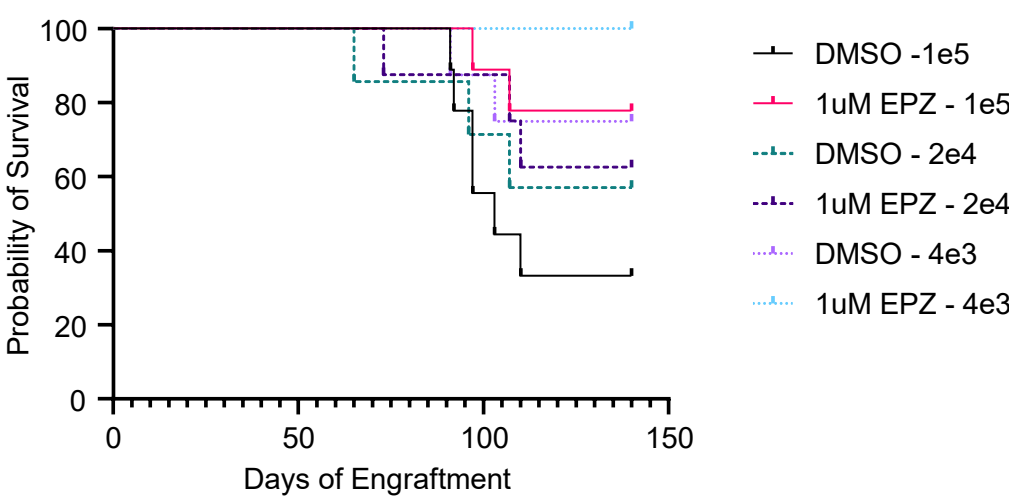

Supplement: Supplementary file 1 [file cancers-16-00569-s001.zip › 20240105 EZH2 Supp Figure S4_Final.pdf]

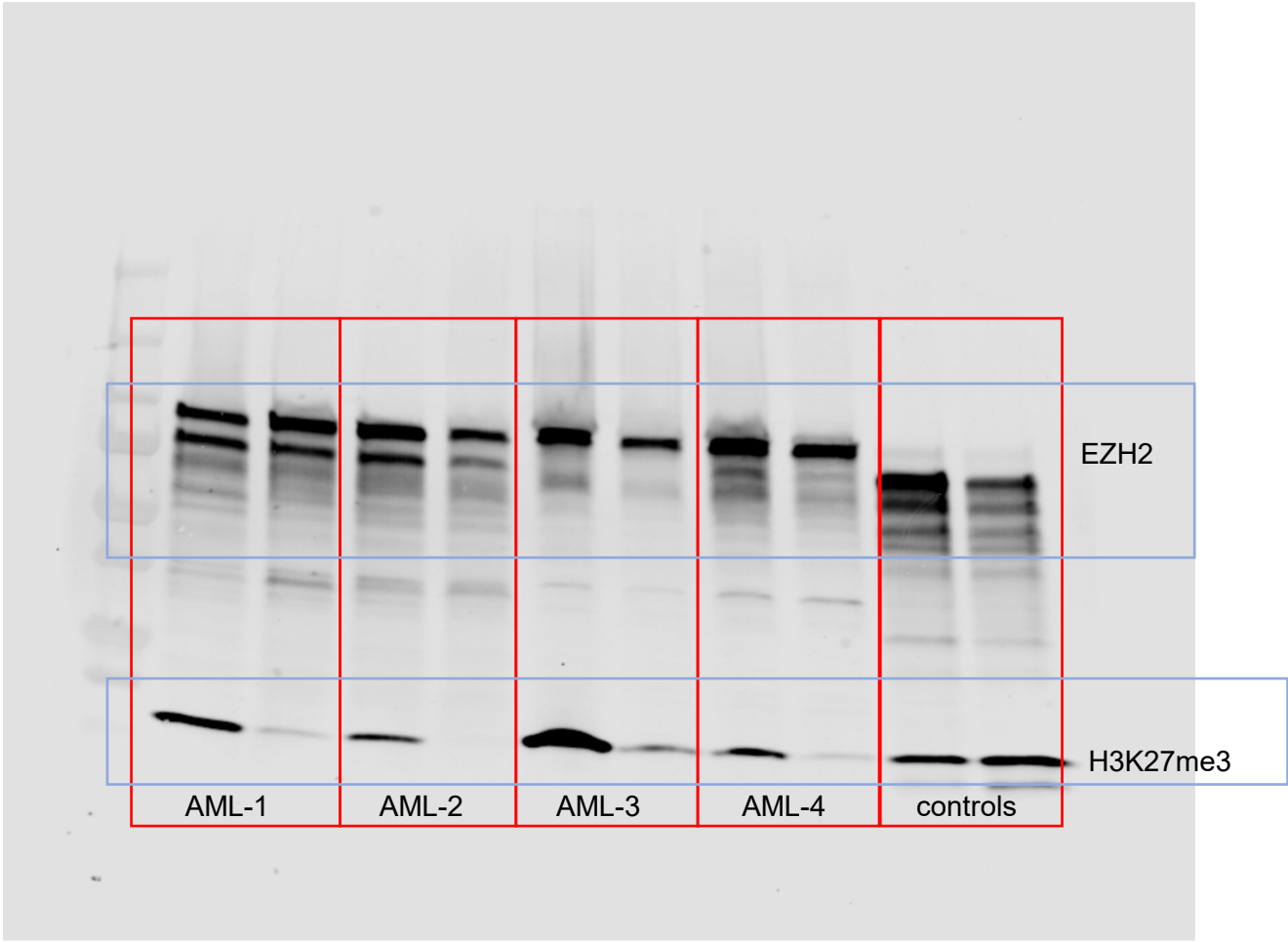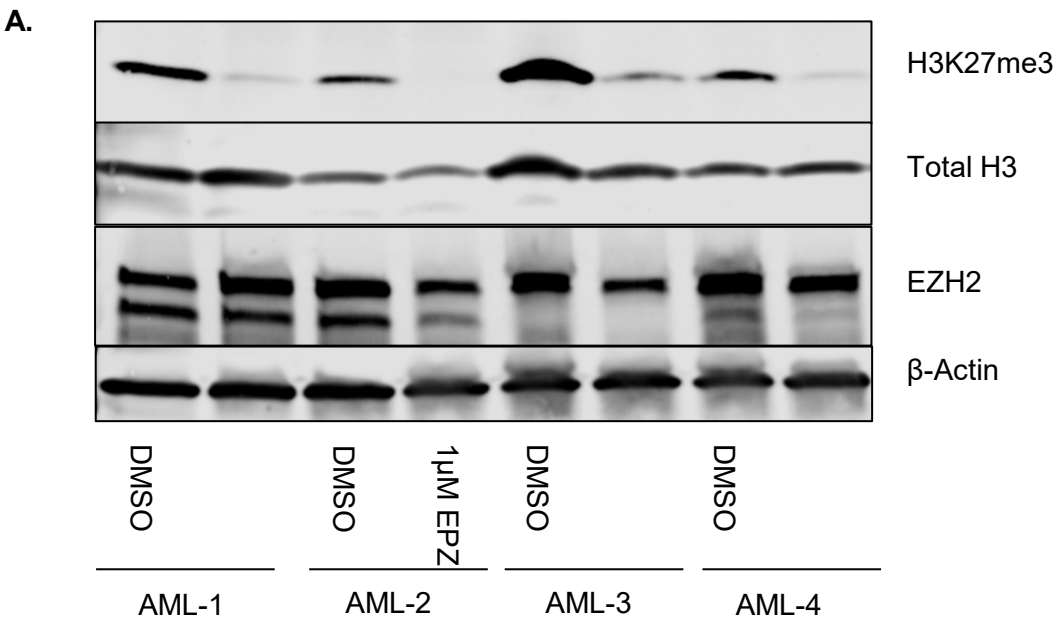

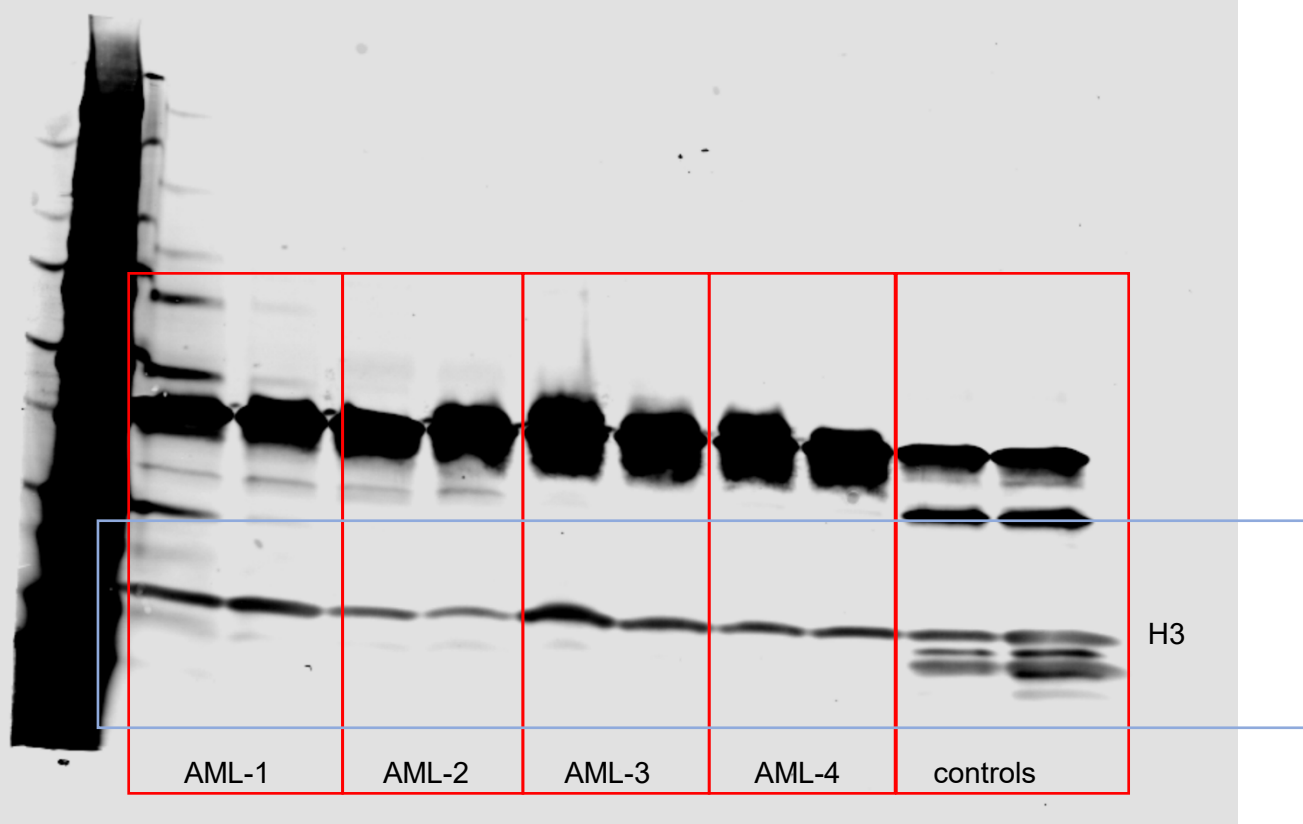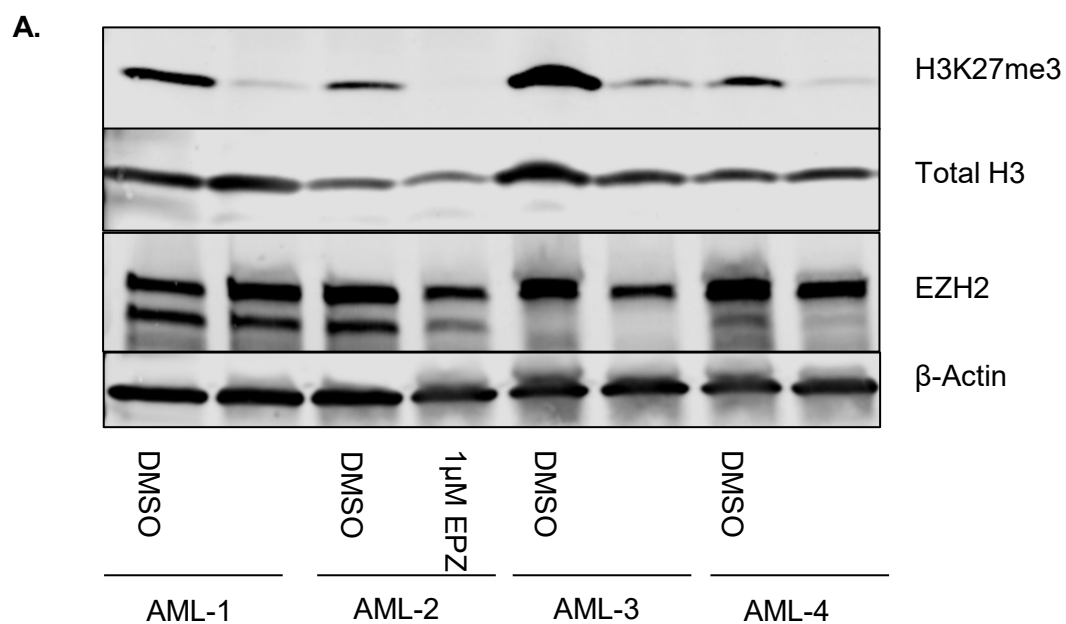

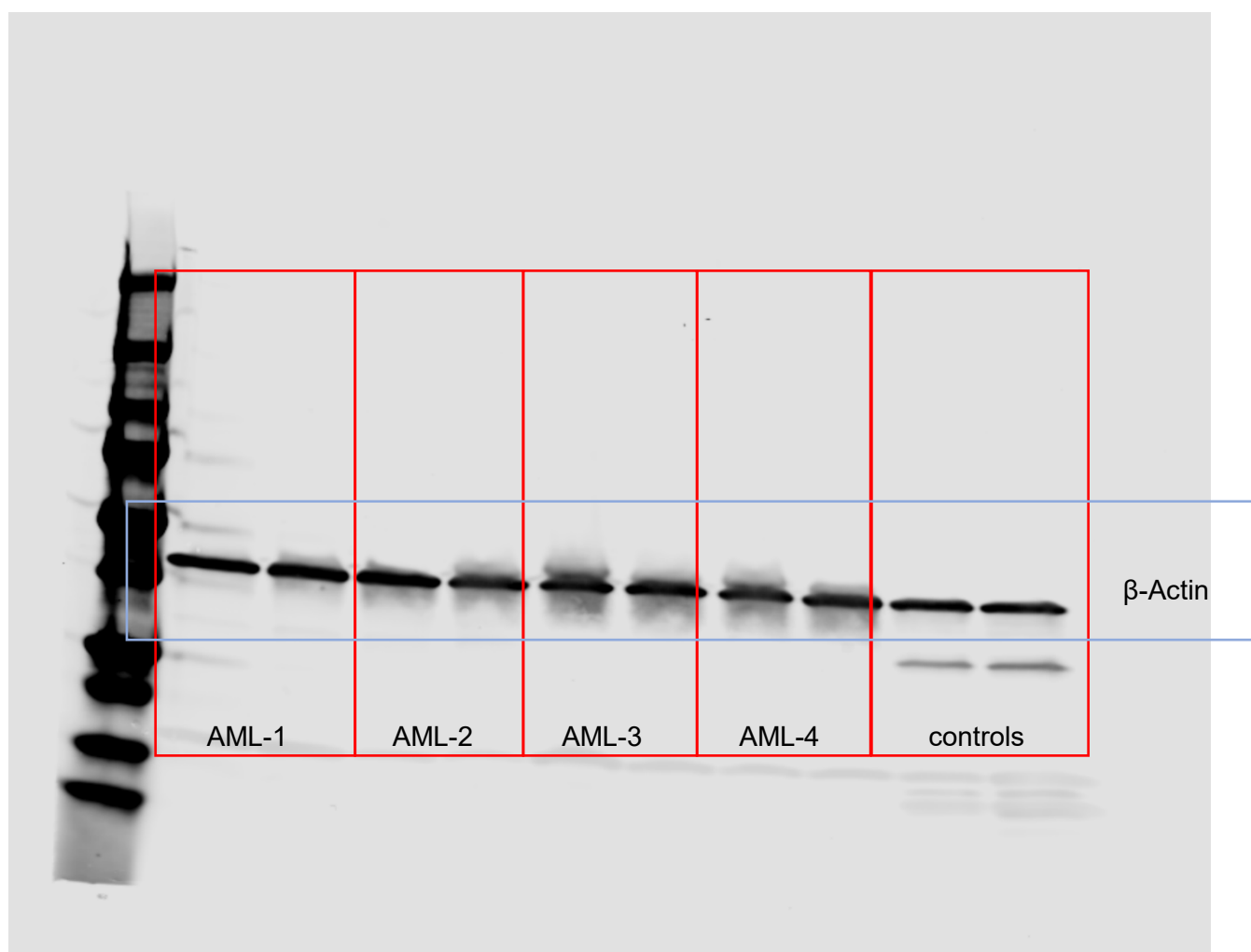

A.

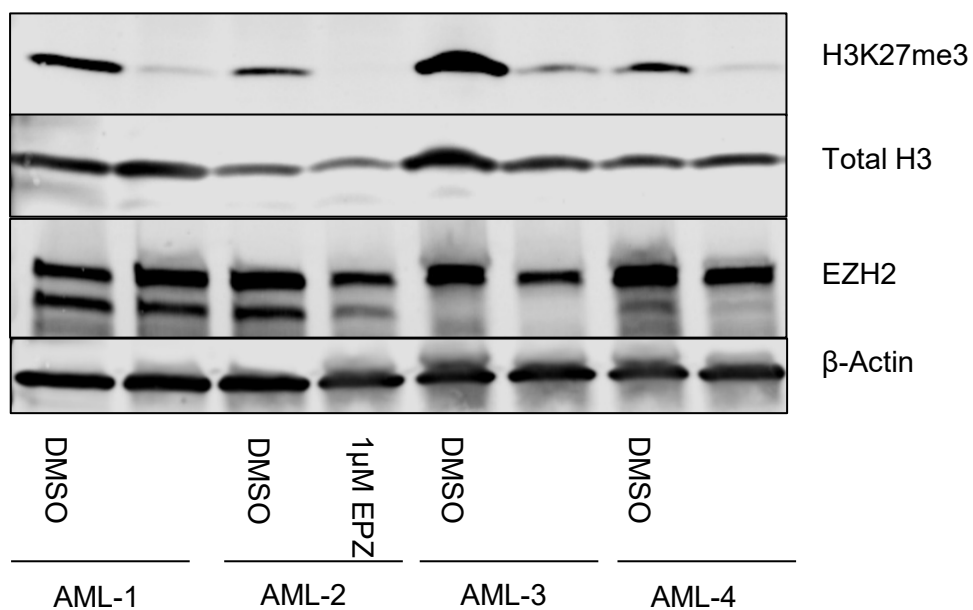

Supplement: Supplementary file 1 [file cancers-16-00569-s001.zip › File S2_uncropped immunoblots for Figure 2.pdf]
